# Pest detection dogs for wood boring longhorn beetles

**DOI:** 10.1038/s41598-021-96450-0

**Published:** 2021-08-19

**Authors:** Charlotte Holmstad Arnesen, Frank Rosell

**Affiliations:** Faculty of Technology, Natural Sciences and Maritime Sciences, Department of Natural Sciences and Environmental Health, University of South-Eastern Norway, 3800 Bø, Telemark Norway

**Keywords:** Zoology, Animal behaviour

## Abstract

Invasive alien species are increasing due to globalization. Their spread has resulted in global economic losses. Asian [*Anoplophora glabripennis* (Motschulsky)] (ALB) and citrus [*A. chinensis* (Forster)] (CLB) longhorn beetles are two introduced wood borers which contribute to these economic losses e.g. the destruction of tree plantations. Early detection is key to reduce the ecological influence alongside the detrimental and expensive eradication. Dogs (*Canis lupus familiaris*) can detect these insects, potentially at an early stage. We trained two privately owned dogs to investigate their use as detection tools. We tested the dog’s ability to discriminate ALB and CLB from native wood borers by carrying out double-blind and randomized experiments in three search conditions; (1) laboratory, (2) semi-field and (3) standardized field. For condition one, a mean sensitivity of 80%, specificity of 95% and accuracy of 92% were achieved. For condition two and three, a mean sensitivity of 88% and 95%, specificity of 94% and 92% and accuracy of 92% and 93% were achieved. We conclude that dogs can detect all types of traces and remains of ALB and CLB and discriminate them from native wood borers and uninfested wood, but further tests on live insects should be initiated.

## Introduction

Invasive alien species are species intentionally or unintentionally introduced by humans outside their natural geographical range^1^. Their presence is likely to cause damage to the economy and environment as they may threaten agriculture, forestry, and the original states of long established native ecosystems and native species^2,3^. Introduction of alien species are increasing with the efficiency, frequency and volume of transportation^4,5^ resulting in global economic losses of hundreds of billions per year^3,6,7^. Globalization in terms of transportation and the trade of goods have compromised natural geographical and ecological barriers between countries and continents through the transportation of species which contributes to their establishment in a new ecosystem^4,8^. Invading species may enter as contaminants on or inside containers^9^, raw logs for saw mills^9,10^, untreated wood for package material^11^ or live plant import^12^.

Asian longhorn beetle (ALB) [*Anoplophora glabripennis* (Motschulsky)] and citrus longhorn beetle (CLB) [*A. chinensis* (Forster)] are two wood boring beetles which can spread through global trade and transportation network from their native countries; China, Korea and Japan^13,14^. While ALB usually spread through untreated wood packing material^11^, CLB mainly spread through live plant import^15^. Usually, the feeding adults do not cause severe damage. The real harm is caused by the subsequent attacks of the wood boring larvae, which eventually kills the host tree by tunneling deep into the heartwood, producing structural weakness and compromising water and nutrient flow^16,17^. CLB generally attack further down the trunk than ALB, usually at ground level or on the roots below ground, or exposed^18,19^. Recently, attacks from these species in European countries such as Italy and France have shown that they prefer hosts in the genera maple (*Acer spp.)*, birch (*Betula spp*.), willow (*Salix spp.),* horse chestnut *(Aesculus spp*.), poplar (*Populus spp.)* and citrus *(Citrus spp.)* (CLB)^19,20^.

Both species are considered highly destructive pests due to their polyphagous (non-fastidious) characteristics^14,20^. They are responsible for an estimated loss of billions of dollars in China^17^ and hundreds of billions in the US^21^. Due to their damaging life cycle, the beetles threaten various forestry sectors including urban forestry^16^, tree nurseries^15^, and agriculture such as fruit orchards and poplar plantations^11,18^. The eradication program of these beetles are both destructive and expensive, and have accustomed both species to quarantine status (emergency measures to prevent introduction and spread of the invasive alien species) in Europe^22–24^.

Studies have shown that the time to complete their life cycle depends on the temperature and their chosen host (in relation to the species and its condition) for ALB^25,26^. Their life cycle varies between one and two years^27^, with two-year cycles often appearing in colder areas^28^. Studies on how temperature effects ALB indicates that the larvae will most likely initiate pupating after two years in northern European countries^25,26^. Early detection is therefore key, and could reduce the extent of the damage as the infestation may not be visually detectable when the larvae is tunneling deep inside of its host^29^.

Several studies have explored different detection methods for these invasive pests. One of these methods includes the use of lure-baited traps containing plant volatiles^30^ or a combinations of plant volatiles and beetle pheromones to attract, detect and trap the beetles^31,32^. Another explored method is acoustics^33^ and laser vibrometry detection^34^, which detects feeding and moving larvae inside its host by using sensors and specialized software calibrated to the feeding larvae`s sound frequency. However, the most frequently used detection method is visual detection, through ground surveys using high contrast binoculars, or tree crown surveys using ladders, hydraulic lifts or trained tree climbers^35^. These human detectors look for noticeable signs and symptoms of infestations such as dieback, adult feeding damage on leaves and branches, exit holes, oviposition pits, oozing sap and frass^18,35^. While ground surveys have proven to be a fast, but uncertain method of checking for infestation, the tree crown surveys are slower and more costly, but with a higher detection rate^26,36^. Since early detection of infestation is crucial to reduce the pests ecological and economic influence, a sensitive and efficient method focusing on non-visual signs of infestation would be of highly importance.

Dogs (*Canis lupus familiaris*) have assisted humans for decades in ecological management, conservation and pest management, through the use of their nose to detect endangered, vulnerable, small and cryptic species alongside their biological traces like feaces, dens and nests^37^. Studies have shown that dogs can be used to detect beetles (*Coleoptera*) such as red palm weevil [*Rhynchophorus ferrugineus* (Olivier)]^38^, hermit beetle [*Osmoderma eremita* (Scopoli)]^39^, bark beetle [*Ips typographus* (Linnaeus)]^40^, ALB^41,42^ and emerald ash beetle [*Agrilus planipennis* (Fairmaire)]^43^. These beetle studies have shown promising results, but some discrepancies that could potentially confound and bias their result were discovered. Unblind handler and test personnel^44,45^, insufficient discrimination training^46,47^, constant target scent presence^48^, non-random sample positions and number of samples present^49^ are all factors that contribute to discrepancies and make the use of detection dogs questionable.

We implemented a testing and training protocol created to minimize bias and confounding results to investigate the potential use of dogs as a tool for the early detection of two invasive alien beetle species. We trained two privately owned dogs to detect all life stages and traces of ALB and CLB, and to discriminate them from native wood borers such as ribbed pine borer [*Rhagium inquisitor* (Linnaeus)], small white-marmorated longicorn [*Monochamus sutor* (Linnaeus)], and small poplar borer [*Saperda populnea* (Linnaeus)]. After the training was completed, we tested the dog`s abilities to detect and discriminate the invasive wood borers from non-target scents such as native wood borers in three different experiments carried out in three different search conditions: (1) laboratory, (2) semi-field, and (3) standardized field. We hypothesized that the dogs would both (1) detect all odors originating from ALB and CLB, and (2) discriminate these from non-target scents such as native wood borers in all three test conditions.

## Results

### Experiment one: scent platform

**Figure 1:**
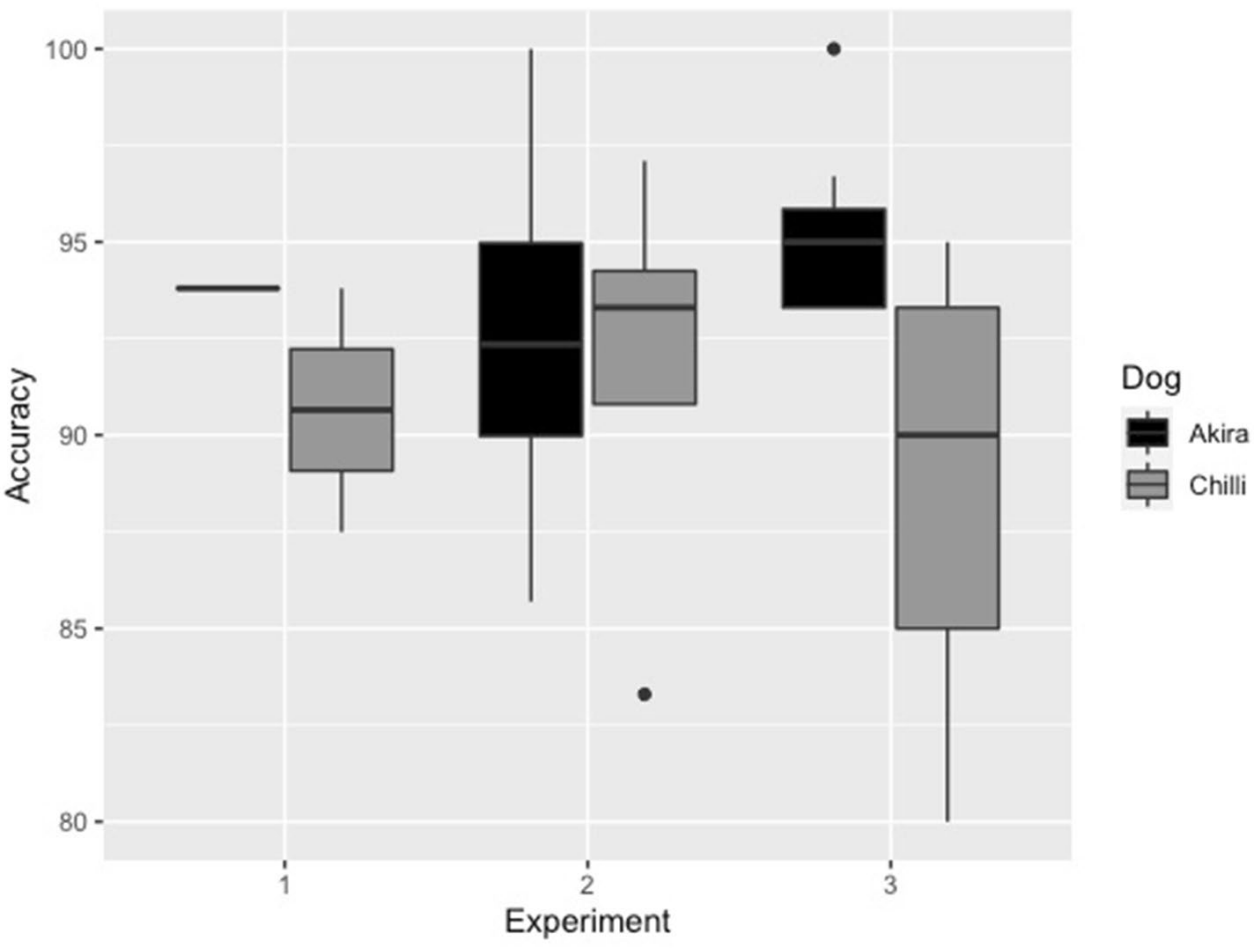
Results presented as accuracy (correct indications among all indication) in percent for all three experiments: laboratory on a scent platform (1), semi-field (2) and field (3).

The two dogs detected ALB and CLB with a mean accuracy of 92.2% (± 3.1 SD) (Fig. 1 and Table 1). Of the 128 scent samples searched, the two dogs falsely detected ALB and CLB five times with a mean, specificity of 95.1 (± 1.9) and falsely rejected ALB and CLB five times with a mean sensitivity of 80.4% (± 9.2 SD) (Fig. 1 and Table 1). The incorrectly identified samples contained frass (three samples), adult (one sample) and larvae (one sample) from ALB and CLB, and frass (one sample), larvae (one samples), adult (two samples) and pupae (one sample) from small white-marmorated longicorn and ribbed pine borer.

**Table 1:** Mean results presented as sensitivity, specificity and accuracy in percent, including ± standard deviation (SD), calculated from 16 randomized and double-blind experimental searches on a scent platform that was carried out between the 17th and 19th of December 2018 at the University of Southeast Norway including both invasive and native wood borers. A dog`s response to a scent sample could be either; *TP* true positive response, *FP* false positive response, *TN* true negative response and *FN* false negative response. *CR* total number of correct responses, *ICR* total number of incorrect responses, and *TOT R* total number of responses.

Dog	TP	FP	TN	FN	CR	ICR	TOT R	Sensitivity (%) ± SD	Specificity (%) ± SD	Accuracy (%) ± SD
Akira	11	2	49	2	60	4	64	84.6 ± 1.7	96.1 ± 0.1	93.8 ± 0
Chilli	10	3	48	3	58	6	64	76.9 ± 13.5	94.1 ± 2.6	90.6 ± 4.4
SUM	21	5	97	5	118	10	128	–	–	–
MEAN	–	–	–	–	–	–	–	80.4 ± 9.2	95.1 ± 1.9	92.2 ± 3.1

### Experiment two: semi-field

The two dogs detected ALB and CLB when searching on trees and untreated wooden pallets indoors with a mean accuracy of 92.2% (± 5.5 SD) (Fig. 1 and Table 2). Of the 274 scent samples searched, the two dogs falsely detected ALB and CLB 11 times with a mean specificity of 94.1% (± 4.2 SD) and falsely rejected ALB and CLB 12 times with a mean sensitivity of 87.8% (± 16.5 SD) (Fig. 1 and Table 2). The incorrectly identified samples contained frass (six samples), larvae (two samples) and adults (four samples) from ALB and CLB, and frass (six samples), adult (one sample) and larvae (four samples) from small poplar borer, ribbed pine borer and small white marmorated longicorn.

**Table 2:** Mean results presented as sensitivity, specificity and accuracy in percent, including ± standard deviation (SD), calculated from 21 randomized and double-blind experimental searches carried out on trees and untreated wooden pallets in a semi-field condition between 11 to 20th of May 2020 at the University of Southeast Norway including both invasive and native wood borers. A dog`s response to a scent sample could be either; *TP* true positive response, *FP* false positive response, *TN* true negative response and *FN* false negative response. *CR* total number of correct responses, *ICR* total number of incorrect responses, and *TOT R* total number of responses.

Dog	TP	FP	TN	FN	CR	ICR	TOT R	Sensitivity (%) ± SD	Specificity (%) ± SD	Accuracy (%) ± SD
Akira	45	7	81	4	126	11	137	92.8 ± 8.3	92.8 ± 6.0	92.6 ± 5.9
Chilli	40	4	85	8	125	12	137	82.8 ± 22.3	95.4 ± 0.9	91.8 ± 5.9
SUM	85	11	166	12	251	23	274	–	–	–
MEAN	–	–	–	–	–	–	–	87.8 ± 16.5	94.1 ± 4.2	92.2 ± 5.5

### Experiment three: standardized field

The two dogs detected ALB and CLB when searching on trees and untreated wooden pallets in the field with a mean accuracy of 93.1% (± 5.8 SD) (Fig. 1 and Table 3). Of the 300 scent samples searched, the dogs falsely detected ALB and CLB 17 times with a mean specificity of 91.6% (± 5.8 SD) and falsely rejected ALB and CLB five with a mean sensitivity of 95.3% (± 10.1 SD) (Fig. 1 and Table 3). The incorrectly identified samples contained frass (two samples), larvae (two samples) and adult (one sample) from ALB and CLB, and frass (ten samples), larvae (four samples) and adults (three samples) from small poplar borer, ribbed pine borer and small white-marmorated longicorn.

**Table 3:** Mean results presented as sensitivity, specificity and accuracy in percent, including ± standard deviation (SD), calculated from 30 randomized and double-blind experimental searches carried out on trees and untreated wooden pallets in field condition between 26th of July to 10th of July 2020 at the University of Southeast Norway and forest areas in close vicinity of the University including both invasive and native wood borers. A dog`s response to a sample could be either; *TP* true positive response, *FP* false positive response, *TN* true negative response and *FN* false negative response. *CR* total number of correct responses, *ICR* total number of incorrect responses, and *TOT R* total number of responses.

Dog	TP	FP	TN	FN	CR	ICR	TOTR	Sensitivity (%) ± SD	Specificity (%) ± SD	Accuracy (%) ± SD
Akira	56	6	87	1	143	7	150	98.5 ± 3.7	93.9 ± 4.9	95.6 ± 2.5
Chilli	52	11	83	4	135	15	150	92.1 ± 13.7	89.3 ± 6.0	90.1 ± 7.2
SUM	108	17	170	5	278	22	300	–	–	–
MEAN	–	–	–	–	–	–	–	95.3 ± 10.1	91.6 ± 5.8	93.1 ± 5.8

## Discussion

According to our hypothesis we have shown that with a testing and training protocol in place that minimizes bias and confounding factors, our dogs were able to detect all traces and remains of ALB and CLB and discriminate them from non-target scents such as native wood borers and uninfested wood. Our dogs were able to do so in three different test scenarios; laboratory with a scent platform, semi-field and standardized field, searching a total of 702 scents (Appendix 2). They falsely rejected 22 target samples (N = 210) and falsely detected 33 scent samples originating from native wood borers (N = 152). They also correctly rejected all scent samples originating from uninfested wood/wood shavings/natural growing trees (N = 186) and blanks (N = 154).

Our results are similar to those from previous studies carried out on beetle scent. The red palm weevil study achieved a mean detection accuracy of 78%^38^ and the hermit beetle study achieved a detection accuracy ranging from 71 to 93%^39^. The Swedish bark beetle study tested dogs in laboratory conditions searching for synthetic bark beetle pheromone. The dogs managed to detect the pheromones 99% of the time and correctly rejected the control samples 55% of the time^40^. The previous ALB study from 2016 achieved an overall detection accuracy of 94%^41^ and the emerald ash borer study achieved a sensitivity ranging from 73.3 to 100% and specificity ranging from 88.9 to 99.8%^43^. These beetle studies have shown promising results, however, some discrepancies that could potentially confound and bias their result were discovered.

A dog`s exceptional ability to interpret intentional and unintentional cues from humans have proven to have an extensive effect on problem solving tasks^50–52^. Dog-handlers and test personnel in vicinity to the dog should be unaware of scent sample ratio^49^, scent sample order^49^ and target scent presence^48^, in order to create a double-blind test design^45^. We followed a strictly double-blind test design for all our olfactory tests, indicating that our dogs did not use human cues to detect ALB and CLB or ignore non-target scents. Unfortunately, the number of target scents present (and material) were known to both dog-handler and test personnel in the previous ALB study^41^ and the emerald ash borer study^43^. Thus, if two target scents were approached early in the test trial, the handler could easily guide the dog to perform TN responses (correctly ignoring non-target scents) for the remaining scents. This was also the case in the red palm weevil study, though the dog-handler and test personnel had only knowledge of the sample ratio^38^. While the dog-handler in the hermit beetle study was blinded, the test personnel was not (single blind)^39^. Finally, the bark beetle study carried out both single and double-blind tests^40^.

Scent generalization is the concept of generalizing between target variations^49,53^. Target scent variations (e.g. different age, reproductive status, diet, population, sex and concentration) enable dogs to identify a scent denominator between targets and is therefore important in scent dog detection work^53,54^. Since ALB and CLB are quarantine species, all signs of their presence is crucial to detect. We therefore exposed our dogs to scent material such as frass (larvae and adult), dead adult beetles, dead larvae, infested wood and eggs. Our results suggest that the dogs were able to detect scents from specimens and even traces of them, even though the scent material differed in age (some ALB frass samples were over 10 years old and stored at room temperature), provided from different specimens and collected from four different laboratories across the world (France, Canada and the USA).

Quarantine restrictions in European countries caused us to use only dead specimens in training and testing. This may affect our dogs in later field searches as the dogs may find it challenging to generalize between live and dead specimens due to the metabolic processes in live insects. However, an Australian study tested if dogs were able to identify live insects when only trained on insect extracts or dead specimens, due to problems such as quarantine restrictions^55^. They trained four dogs using either insect extract or dead specimens from the bronze orange bug [*Musgraveia sulciventris* (Staal)] to test whether the dogs could detect live insects in the final olfactory test. The dogs successfully detected live insects in the final test with a mean sensitivity of 100% and 75%^55^. The headspace chromatograms of the scent extraction and the dead specimens showed VOCs similarities of 71% and 77% with live insects^55^ indicating that live and dead insect may to some extent excrete similar scents and dogs may generalize between them. A Taiwanese study carried out on scent detection dogs and the red imported fire ant [*Solenopsis invicta* (Buren)] used dead specimens in the preliminary training, before further training and testing with live specimens without any problems^56^, also indicating scent similarities between live and dead ants. However, some scent detection dogs do discriminate between live and dead insects to detect only ongoing infestations, such as bed bug [*Cimex lectularius* (Linnaeus)] detection dogs^57,58^. Nevertheless, all signs of the insect pests (dead or alive) are important to detect since Norway (or the majority of Europeans countries) do not have an establishment of the species; therefore any detection would be valuable to lessen the expensive and detrimental eradication programs.

A large sample size goes hand in hand with enabling dogs to detect the target`s scent denominator. A UK study discussed the memory of dogs, as their dogs possibly remembered 117 unique training scent samples, their results showed an unmistakable decline when 124 naïve scent samples were used in a final olfactory test^54^. Therefore, a large sample size will increase the probability of a detection dog to learn the target`s signature odor^53,54^. A limited sample size (e.g. pseudo-replication of individuals, one type of scent material, one population or one area) will increase the chances of the dog remembering specific samples^54^. We therefore implemented a large sample size (N = 420, target = 210, non-target = 210) and used naïve samples for the three olfactory tests to decrease the chance of the dogs remembering specific samples. We also changed the training samples with new material/individuals at least twice during the training period. None of the previous beetle studies mentioned the sample size or if naïve scent samples were used in the final olfactory tests.

A dog`s highly sensitive nose has an unique ability to discriminate between species, diseases and biological and chemical compounds^37^. The difficulty of the discrimination training should be evaluated based on the deployment of the detection dog. In laboratory conditions the researcher can control which scents the detection dog may encounter and in field conditions the researcher cannot. Specificity is crucial in situations where the target`s presence may play an important ecological or economic role in conservation of vulnerable/endangered species, or the eradication of invasive species. It was therefore of high importance to prepare the dogs for scents they could potentially encounter in the field such as frass, larvae, or adult beetles from other longicorns (*Cerambycidae*). High quality discrimination training can potentially prevent further species identification in laboratories as the result in the field is immediate. The bark beetle, red palm weevil and the previous ALB study did not use non-targets scents they could have encountered in field^39–41^. The previous ALB study used empty paper sheets or uninfested wood as non-target scents in their two test conditions^41^. They did report that in the old orchard, nine of 14 dogs falsely indicated the presence of ALB, but further discussed that the aim was not to test their dog`s discrimination abilities, but rather their ability to detect ALB^41^. Nevertheless, they achieved a high sensitivity (75–88.1%) and specificity (85.3–95.6%) under standardized field tests searching for ALB^41^. In the emerald ash borer study, they used trees attacked by the fungus ash dieback (*Hymenoscyphus fraxineus*) as non-target scents for one of the seven experimental set ups, while in the rest of the experiments they used logs, fire woods, ash trees and other deciduous trees as non-target scents. The hermit beetle study did include non-target scents from species closely related to the hermit beetle in training and testing, but unfortunately stopped the preliminary discrimination training as they experienced higher error rates^39^. However, in the final tests non-target scents were included and the dog showed a lower sensitivity (29%) towards flower chafer species compared to the hermit beetle (69%) and specificity from the tests including natural growing trees ranged from 73 to 92%.

Dogs have proven to be an effective detection tool with both a higher^59–61^ and faster^60,62^ detection rate than humans. The revisiting of areas to collect hair, insects or video footage from hair, pheromone or camera traps are removed since dogs only need one sweep around the search area to disprove or confirm the targets presence^63^. The use of dogs can also reduce search bias, as they are highly oriented on olfaction cues, rather than convenient search paths or visual cues^53^. While human detectors may need to bring specimens to the laboratory for further identification and may not be able to discriminate symptoms (e.g., exit-holes, frass or oviposition pits) from other similar species, dogs with proper discrimination training may potentially identify their presence immediately as they are trained to ignore other wood borers with a similar ecology and morphology in addition to detect all life stages from egg to adults and their frass or infested wood.

Successfully training detection dogs can be expensive and time consuming from providing and preparing samples to the dogs training, testing and maintenance. However, when you have already found a suitable detection dog, additional target scents can be learned. Williams and Johnston^64^ showed that dogs could detect and discriminate 10 target odors from non-targets with a high detection rate within a short time frame in laboratory conditions. Such dogs can also detect several trained target animals within one search^65,66^. Furthermore, extinction training (training on not to indicate the presence of previous conditioned targets^67^) can also be implemented on previous targets that has lost its importance for the researcher. Orkin et al.^68^ suggested that partnership with public agencies or the police could reduce the cost of training and deploying detection dogs as they have been previously used for scent detection.

Individual differences are often seen between scent detection dogs, as some may be more suitable than others in relation to training drive, age or physical condition^69^. No tests were carried out to compare the results of the two detection dogs, however, Akira achieved slightly higher results in all three experiments. Several factors can be the reason for this, such as age and favorable wind/temperature conditions. There is an age difference of eight years between Chilli and Akira, and Chilli was 13 years old when the study was finished. Even though the olfactory system may not be influenced by age after dogs are 14 years old^70^, it may be by physical condition. Factors such as varying terrain, temperatures and working time may potentially have affected the physical exertion of Chilli and influenced her detection abilities due to e.g. excessive panting. Akira may also have experienced more favorable field conditions in relation to search strategies, environmental conditions, and detection distance such as e.g. higher wind speed, lower temperature and higher relative humidity (Savidge et al. 2011; Reed et al. 2011; Glen et.al. 2018). Akira did more frequently use wind currents to navigate to the odor source due to higher wind speed than Chilli.

When it comes to the numbers of dogs used in the respective study, our numbers were limited. However, we did score two out of three possible points on the criteria list regarding number of dogs used in scent detection dog studies, based on a previous study on how to identify bias in scent detection dogs^49^. It is also important to state that our dogs were privately-owned and not professional working dogs^71^. However, the usage of privately-owned dogs contrary to professional working dogs should imply that majority of dogs should be able to achieve similar research outcomes following the given training and test protocol.

We show that our dogs were able to detect all types of traces and remains of the ALB and CLB and discriminate it from native wood borers and other non-target scents such as uninfested wood. However, due to quarantine restrictions, tests were only carried out on dead specimens, and further testing including live insects should be initiated. We therefore conclude that dogs can be used as a detection tool for remains of ALB and CLB and tree symptoms caused by them such as frass and infested wood and be deployed as detection dogs in the field, preferably in addition to current detection methods.

## Methods

### Scent samples

Scent samples (N = 210) from ALB and CLB were provided from Animal and Plant Health Inspection Service (USDA-APHIS), Insect Production Service (IPS, Canada), Forest Service (FS, USDA) and European Biological Control Laboratory (USDA-EBCL, France), where the beetles were reared in laboratory conditions (Appendix 1). Scent samples from FS were collected during a quarantine period in 2010, while the remaining scent samples were collected in September–November 2018. All samples were frozen when killed and/or collected, and were still frozen upon arrival by air mail (except the 10-year old frass samples from FS). Adults were reared on *Acer spp*. and *Salix spp.,* while larvae were reared on either similar diets as the adults or an artificial diet. Dead animals were used because of quarantine restrictions and import challenges of invasive alien species into Norway^22,23^.

Scent materials were handled with sterilized equipment such as tweezers and disposable gloves and separated by species, stage of life cycle (egg, larvae and adult) and type of traces (frass from larvae or adult and infested wood). Frass and infested wood were partitioned into glass jars with teflon lids (30 ml, 57 × 27.5 mm, QORPARK, USA) by weight (AND Electronic balance FA-2000, AC Adapter DC 12 V 0.3A, Norway) (mean = 2.26 g ± 0.3 SD), while larvae, adults and eggs were separated into glass jars by individuals (some early developed larvae and eggs were separated by 1–7 individuals depending on their size) before frozen (− 20 °C). Half of the samples (105) were used in training, whereas the remaining (105) was used in experimental testing to ensure all samples in the final testing were naïve to the dogs^54^. Scent sample material used during training were replaced 1–3 times during the entire training period to avoid familiarizing to specific specimens.

To assure a correct odor impression and decrease the chance of dogs detecting non-target scents during search situations, scents naturally occurring in real-search scenarios were added as controls (N = 210)^46,47^. Environmental non-targets assumed to be easy for the dogs to discriminate from target scents were uninfested wood shavings, bark and human-made sawdust from preferred host trees [goat willow (*S. caprea),* Norway maple *(A platanoides),* white birch *(B. pubescence),* European ash *(Fraxinus excelsior),* European aspen *(P. tremula),* grey alder (*Alnus incana)* and European mountain ash *(Sorbus aucuparia)*]^19,20^ with no visual signs of infestation of wood borers. Uninfested wood shavings and bark were collected in broadleaf forestry areas around the University of South-Eastern Norway, Bø in Midt-Telemark municipality, while sawdust was made by sawing branches off preferred host species and collecting it. Non-targets assumed to be more challenging to discriminate from target, were infested wood, frass, larvae, pupae and adults from native wood borers, such as ribbed pine borer, small white-marmorated longicorn and small poplar borer. Those samples were collected in different broadleaf and coniferous areas within Midt-Telemark municipality. All samples were prepared similarly as the target samples (sterilized equipment and sample glasses), and half of the non-target samples were used during training (105) and the remaining (105) were used in the experimental testing. Empty sample glasses (phase one), disposable gloves (phase one), and blanks (nothing) (all training phases and experiments) were also used as non-targets.

### Dog training and olfactory experiments

Two privately owned female dogs, grosspitz and border collie of three and 11 years old at start respectively, were used in this study. Both dogs had been used in previous scent detection work on beavers (*Castor spp.)*^72,73^ and grouse (*Lagopus lagopus and L. muta*)^74^. One female handler with some experience^74^ trained and handled both dogs.

Training was carried out at the dog laboratory at the University of South-Eastern Norway, Bø in Vestfold and Telemark county, and in forest areas around Bø. The dogs were trained during three periods instead of one continuous due to financial reasons: (1) 1^st^ of October to 21^st^ of December 2018, (2) 8th of January to 13th of June 2019 and (3) 8th of January to 25th of June 2020. The dogs were periodically trained within the respective time frames (0–3 sessions a week), and active training time ranged from 10 to 30 min for each dog in a session. We used 100% positive reinforcement training with operant conditioning using a clicker^75,76^. Food, praise and/or play was used as a reward. The training program was split into three phases: (1) target imprinting on a scent platform in the laboratory, (2) semi-field training and (3) standardized field training. A double-blind experiment was carried out after each training phase (i.e., three experiments) to ensure reliable and consistent detection.

#### Phase one: target imprinting on scent platform

**Figure 2:**
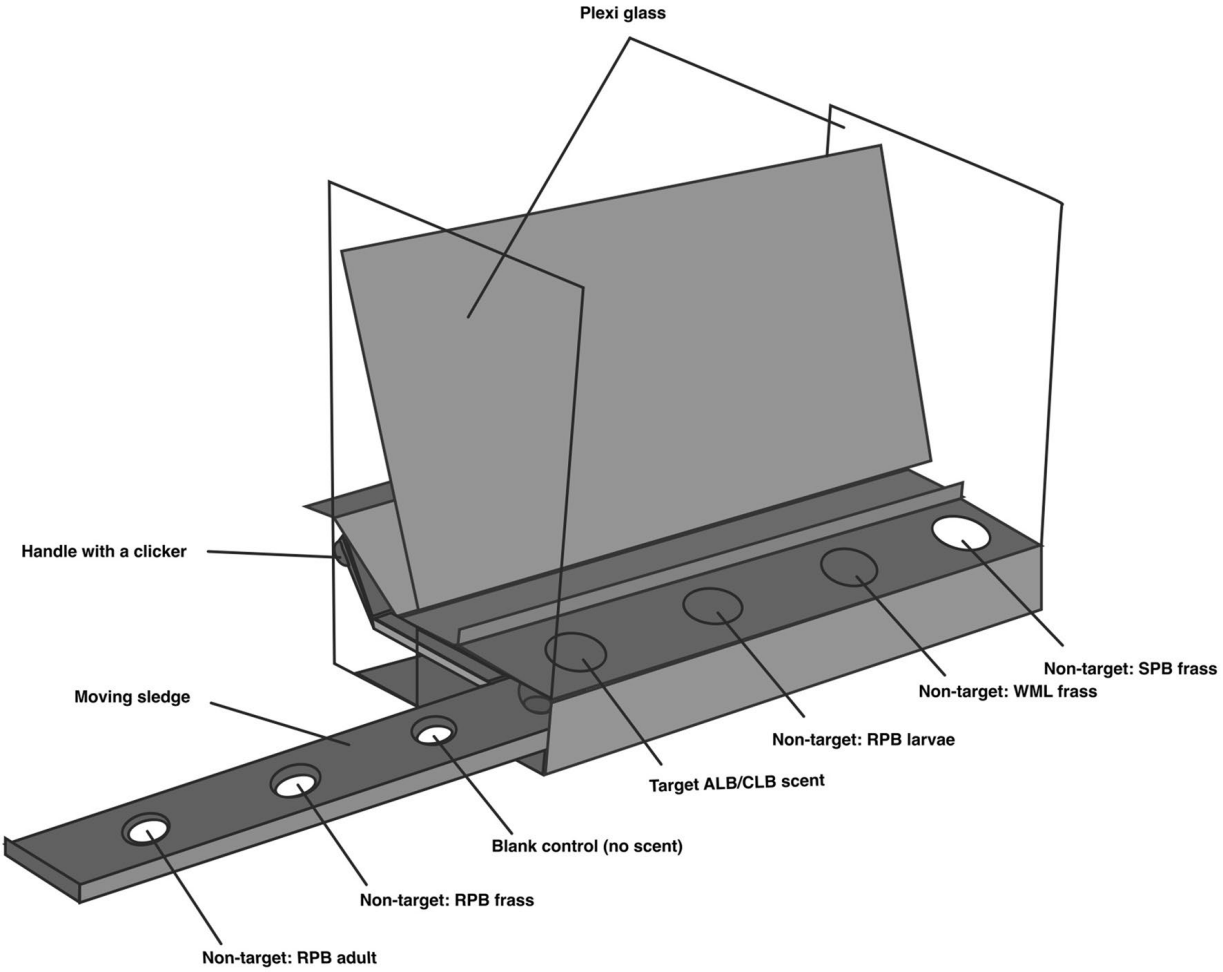
Table platform used in training and experiments one. All training and experimental trials involved a random sample layout and could contain additional non-target scents as well. Target: ALB [*Anaplophora glabripennis* (Motschulsky)] or CLB [*A. chinensis* (Forster)]. Non-target: small white-marmorated longicorn (WML) [*Monochamos sutor* (Linnaeus)], ribbed pine borer (RPB) [*Rhagium inquisitor* (Linnaeus) small poplar borer (SPB) [*Saperda populnea* (Linnaeus)], and non-infested wood from *Salix spp*. and *Acer spp.*

A standard table platform adapted by Hundcampus Training Centre (HÄLLEFORS, Sweden) was used in phase one (Fig. 2). The rationale behind this scent platform is that one target scent is always present among three non-target scents (the platform holds a total of seven scent holes, one target scent and six non-target scents) (Fig. 2). Thus, the dog will learn to discriminate the target scent from the other non-target scents.

Responses to a scent sample in the scent platform was registered and used to calculate training and test statistics. Both dogs had been used in previous studies with similar initial training and taught to independently investigate a platform and lay down in front of a target sample to indicate its position for 3–5 s. When a dog was presented with a scent sample, it could respond in four different ways: 1) false positive (FP) response, the dog is performing a trained response on non-target sample, 2) true positive (TP) response, the dog is performing a trained response on a target sample, 3) false negative (FN) response, the dog falsely rejects a target sample and (4) true negative (TN) response, the dog correctly rejects a non-target scent^77,78^.

Phase one started with target imprinting on the scent platform. We used one glass jar containing a target sample (frass from larvae, frass from adult, wood shavings, larvae, adult or eggs from ALB or CLB) placed in a plastic cup among six non-target scents (disposable glove and empty sample glass) also placed inside a plastic cup or blanks (nothing) (Fig. 2). We used disposable gloves and new plastic cups for each scent to avoid scent contamination between target and non-target scents and the platform. To imprint the dogs with the target scent, two training sessions per target material were carried out and all indications on the target material was immediately rewarded with a click from the clicker and a food/praise reward. An accuracy level of ≥ 80% in two consecutive sessions was required before training with all variations of the target. One training session consisted of five searches where the handler rapidly changed target position ten times (i.e., a dog searched 200 scent samples per training session). When the dogs had imprinted on all types of target scents, the next training level was initiated.

The next training level within phase one included discrimination training. First, environmental non-target scents (uninfested wood shavings, human-made sawdust and bark from preferred host species) were added, and when the dogs achieved an accuracy of ≥ 80% in two consecutive sessions, scents from native wood borers (ribbed pine borer, small white-marmorated longicorn and small poplar borer) were added. Experiment one was initiated after achieving an accuracy of ≥ 80% in two consecutive sessions in the discrimination training.

#### Experiment one: standard scent platform

The dogs were tested to see whether they could discriminate all life stages and traces of ALB and CLB from all types of control species and uninfested wood (non-target scents) using the scent platform. Experiment one (consisting of two experimental sessions) was carried out the 17th and 19th of December 2018. Contrary to phase one, the structure of a session was changed, and the dogs had to search the platform eight times per experimental session (i.e., a dog searched 32 scent samples per experimental session, thus a total of 64 scent samples was searched per dog in experiment one). To avoid as much interaction and unintentional cueing between the dog and the handler as possible, the handler was placed two meters in front of the platform (behind the dog), sending the dog to independently smell the platform^79^.

An experimenter placed the scent samples within the platform to ensure the experiments were blinded^45,49^. As in training phase one, only one target sample could be present in a search, but empty searches were included to ensure that the handler was unaware of the target presence, hence no unintentional cueing from the handler to the dog^51^. Target presence (and its position in the platform) was further randomized each search using a random number generator so the handler was totally unaware of the sample ratio and unable to cue the dog^48,49^.

After the scent samples were placed within the platform, the experimenter exited the training room, and the dog-handler team entered the training room (i.e., the experimenter and the dog-handler team were in separate rooms). In the second room, a video monitor connected to cameras allowed the experimenter to watch the dog-handler team live, without the ability to cue the dog-handler team, i.e., double blind test design^45^. The experimenter was equipped with a clicker that could be heard from across the two rooms. The clicker was used as a tool to validate the team’s response, and a click was provided if the response was correct (TP or TN) and withheld if incorrect response was made (FP or FN). We were therefore able to avoid target confusion (often associated with always reinforcing and rewarding each indication regardless of its correctness) and loss of confidence (often associated with always withholding reinforcement and rewards regardless of the indication`s correctness) in the experimental design^54,80^.

All experimental searches were recorded with two cameras (SONY HANDYCAM DCR-SR, Norway) filming the team`s responses. All recordings were watched and responses validated by an independent observer to avoid observation bias^81^. When both experimental sessions were completed, phase two was initiated.

#### Phase two: semi-field training

Phase two was designed as an incremental step to prepare the dogs for field searches by an environment simulating more closely field conditions, while still remaining in familiar surroundings of the laboratory. Trees and untreated wooden pallets were now used as search object, contrary to the scent platform used in phase one.

**Figure 3:**
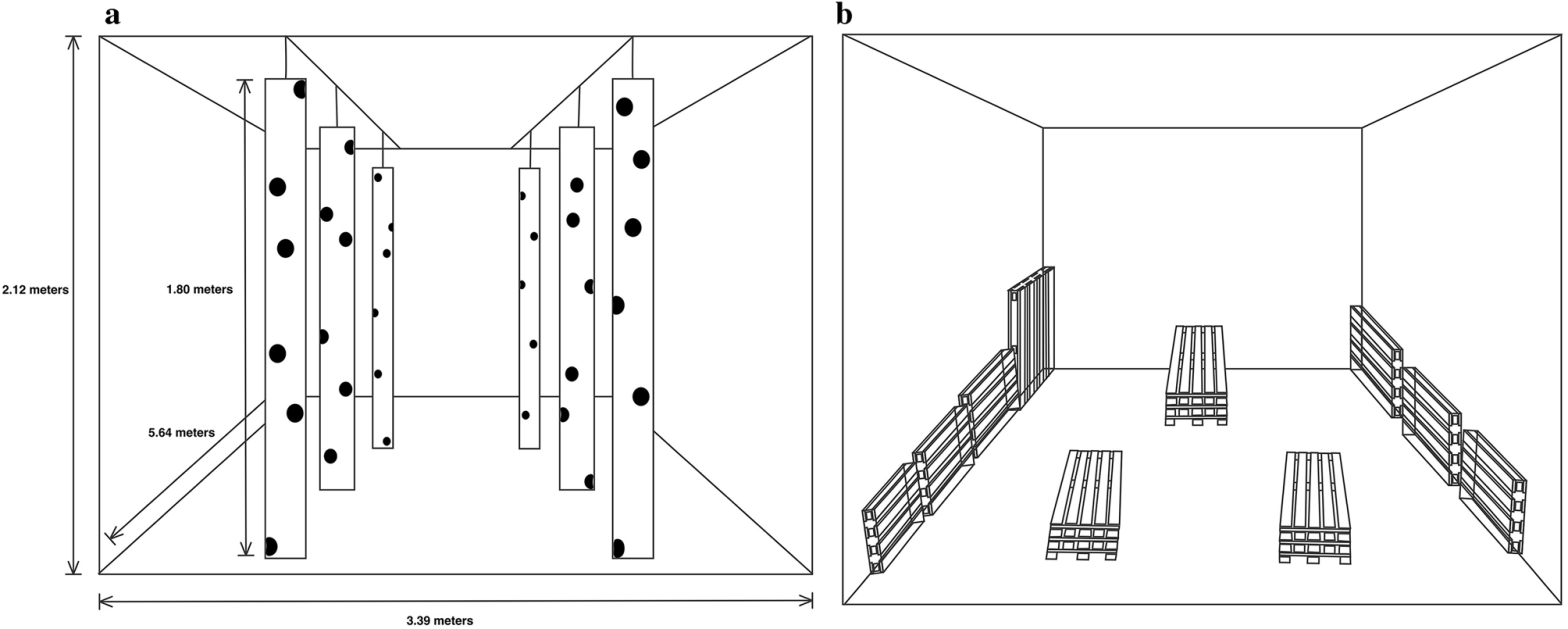
(**a**) The laboratory during the semi-field condition and experiment two. Six truncated trees [European aspen (*Populus tremula*), European mountain ash (*Sorbus excelsior*), European ash (*Fraxinus excelsior)*, Norway maple (*Acer platanoides*), goat willow (*Salix caprea*), white birch (*Betula pubescence*) and grey alder (*Alnus incana*)] were drilled with 7–10 scent holes and hanged down from two railing systems. The trees could be moved alongside the railing systems. (**b**) The laboratory during semi-field condition and experiment two. Seven pallets stacked against the wall or three stacks of three pallets.

Six truncated trees (height: 1.8 m, radius: > 7.5 cm, weight: < 20 kg, species: European aspen, European mountain ash, European ash, Norway maple, goat willow, white birch and grey alder) were drilled with 7–10 holes per tree (scent jar measures: circumference = 12 cm and depth = 7.5 cm) at a varying height (from directly above ground to 1.8 m) (Fig. 3a). The scent jar openings were covered with unscented parafilm (2 in. × 250 ft. roll, BEMIS, USA) that later were perforated to allow scent molecules to exit the parafilm lid. Perforated parafilm lids were chosen to minimize the possibility of scent dissipating onto the trees when the jars were placed within the drilled cavities, which also made it possible to reuse the trees. The truncated trees were hung down from two railing systems on the roof of the laboratory (Fig. 3a). Each tree could be moved throughout the length of the rail; hence, the trees could be moved to different locations alongside the railing system.

Phase three started with training the dogs to pinpoint target positions on different objects than the scent platform, i.e. trees. First, target samples were placed inside cavities at a similar height to the dog´s head. The dogs were told to search while the handler pointed at the target position, clicking with the clicker immediately when they sniffed the target. Soon they understood that pinpointing the target position with their nose would result in a click and a reward. Distance from the dog to the target scent gradually increased, which enabled the dogs to follow the odor source^40^. Target samples were now placed at varying height, from ground level up to 1.8 m, corresponding with the ALBs and CLBs general attack pattern^20,25,82^.

When the dogs were able to pinpoint target positions at varying heights, the semi-field training continued. A training session consisted of six searches, i.e., a dog searched 36 trees per training session. Non-target samples and empty searches (searchers with no target sample present) were included so the dogs would still train on discriminating target scents from non-target scents. This should increase their detection reliability, as no indications should be given if the target scent was absent^48^. Number of target and non-target scents per search varied and sample position was always randomized (MICROSOFT EXCEL, version: 16.16.3, 2016)^49^.

Every third training session, the dogs searched stacks of wooden pallets (three stacks of three pallets on top of each other) or pallet bedded walls (seven pallets; three and four pallets per long side of the wall) (Fig. 3b). The parafilm-covered scent jars were placed within the pallets in different hiding places to imitate beetle-attack in untreated wood packing material^11^. Six searches were carried out per training session, either on the three pallet stacks, i.e., a dog searched 18 stacks of wooden pallets per training session, or the seven wall-bedded pallets, i.e., a dog searched 42 wooden pallets per training session. As with the tree searches, the number of target and non-target scents varied, as did sample position^49^. When the dogs achieved an accuracy of ≥ 80% experiment two was carried out.

#### Experiment two: semi-field

The dogs were tested to see whether they could discriminate all life stages and traces of ALB and CLB from all types of control species and uninfested wood (non-target scents) in a semi-field condition searching on trees and untreated wooden pallets indoor. Experiment two (consisting of four experimental sessions; two sessions searching on pallet bedded walls and two sessions searching on trees) was carried out between 11 and 20th of May 2020.

Pallet and tree searches were carried out in the same manner as in phase two. Originally, an experimental session consisted of six searches of either the seven pallets or the six trees, but because both dogs experienced fatigue and lack of motivation during the last search, we readjusted the number of searches in the remaining experimental sessions to consist of five searches instead of six. Accordingly, a dog searched 35 pallets or 30 trees per experimental session (except for the first experimental session where the dogs searched 42 pallets each). We further followed the same procedures as in experiment one. When the four experimental sessions were completed, phase three was initiated.

#### Phase three: standardized field training

Training was carried out in forest areas searching on trees, or in close vicinity to the dog laboratory searching on stacks of wooden pallets. The time used to initiate this phase was controlled by factors such as finance (three training periods), detection accuracy (≥ 80% accuracy in two consecutive training sessions) and environmental factors (wind speed (m/s), temperature (C°), precipitation (mm) and snow). Training was only carried out with wind speed < 5.0 m/s, temperatures between 0 and 29 °C, precipitation < 1.5 mm and in addition the absence of snow. These weather factors were based on previous findings on search strategies, environmental factors and detection distance for detection dogs^83–88^ and measured and registered before each training session. Terrain could vary from flat to very steep (flat, moderate, or very steep), temperature from 0 to 29 °C, precipitation from 0 to 1.5 mm and wind speed from 0 to 5.0 m/s.

Tree searches were carried out in forest areas with a heterogeneous species compositions due to the ALBs and CLBs polyphagous characteristics^14,20^. Forest areas were chosen based on tree availability and search scenario (operative industry areas, urban areas, or busy roads). All scent materials were inserted into small individual filter bags (tea bags, 50 × 70 mm) to ease placement and hiding on branches, inside cracks in the bark at different heights or under exposed roots to imitate beetle attacks^11,18,19^. As the filter bags were hidden, no visual cues could be followed from neither the dog nor the handler^52^. All trees searched were walked up to, in a random order, and touched by the person placing the scent samples before each search, so no scent trails could be followed to a specific scent sample^49,89^. One training session consisted of six searches on six trees (i.e., a dog searched 36 trees per training session), and to avoid familiarization, residual scent and scent trails, the same areas were never revisited before at least 3 weeks^39^. Dogs were on a tracking leash while searching, and the handler did no corrections using the leash, but simply guided the search so the dogs sniffed each tree in the trial while looking for indication behaviors.

Every third training session, the dog searched stacks of pallets outside near the laboratory. The training design was equal to the indoor training on pallets, but the dogs were now only searching on stacks of pallets (two stacks of five pallets), i.e., each training sessions consisted of six searches. Contrary to the tree search, all scent material was kept inside the sample jars as in the previous indoor training, using perforated parafilm lids, so the same pallets could be reused with minimal chance of residual scent. Experiment three began when the field training was completed (accuracy of ≥ 80% in three consecutive sessions).

#### Experiment three: standardized field

The dogs were tested to see whether they could discriminate all life stages and traces of ALB and CLB from all types of control species and uninfested wood (non-target scents) in field condition searching on trees and untreated wooden pallets outside. Experiment three (consisting of six experimental sessions; three sessions searching on stacks of pallets and three sessions searching on trees) was carried out between 30th of June and 20th of July 2020.

The experiments took place at areas previously used in training, but to decrease the chance of residual target/non-target scent, we used at least 3 weeks in between a search.

Experiment three was carried out in the same manner as phase three. Either two stacks of wooden pallets or six naturally growing trees were searched five times per experimental session, i.e., a dog searched 10 stacks of pallets or 30 trees per experimental session. Standardization of the search areas were done by marking the six trees that were to be searched with colored ribbons, so the dog handler knew which trees the dog needed to investigate for each search. The experimenter and dog-handler went through the order of trees together before a search. Further, the same experimental design as experiment one and two was used.

A search object (a tree or a stack of pallet) could in this experiment contain either one target scent, one non-target scent or no scent (natural growing tree as non-target scent) or two target scents, two non-target scents or two blanks (one stack of pallet was divided into two (left and right side of the stack)). After the scent samples were placed within the trees/pallets, the experimenter visually exited the search area and the dog-handler team`s responses were now verified by telephone between the dog handler and the experimenter. A chest-mounted video camera (GOPRO HERO 5, 445222, Norway) recorded each search.

Environmental conditions were registered in the beginning and end of each experimental session, taking the average as measurements of temperature (TESTO SAVERIS 2, Norway), wind speed (EXTECH AN25, Norway) and relative humidity (%) (TESTO SAVERIS 2, Norway). Data on how many days since precipitation (0–1 days) was retrieved from a local weather station^90^. All the field experiments were carried out in either sunny and cloudless weather, cloudy weather, or light rain (< 3 mm). Light rain was experienced in a total of nine experimental sessions (three for Chilli and six sessions for Akira) (Table 4). Only Chilli experienced experimental sessions without precipitation. Wind speed ranged from 0 to 4 m/s with a mean wind speed of 0.75 (± 1.26 SD) m/s (Table 4). Temperature ranged from 14.4 to 25.5 °C with a mean temperature of 18.7 °C (± 3.4 SD) (Table 4). Relative humidity ranged from 45.1 to 95.2% with a mean percentage of 65.8% (± 17.5 SD) (Table 4).

**Table 4:** Environmental variables during 30 randomized and double-blind experimental searches carried out on trees and untreated wooden pallets in field. The environmental variables include temperature (°C), wind speed (m/s), relative humidity (%) and days since precipitation (> 3 mm), and represents mean (± standard deviation, (SD)) and range of measures for all the 30 experimental searches.

Dog	Winds speed (m/s)	Temperature (°C)	Relative humidity (%)	Weather	Days since precipitation
Akira	1.2	21.0	48.9	Light rain	0
	0	22.4	45.1	Light rain	0
	4	21.1	95.2	Light rain	0
	2.3	18.2	78.9	Light rain	0
	1.3	25.5	64.7	Light rain	0
	0	16.6	93.4	Light rain	0
Chilli	1.1	15.2	75	Cloudy	1
	0	14.4	61.3	Cloudy	0
	0	15.2	73.9	Cloudy	0
	0	15.7	54.7	Cloudy	1
	0.1	19.8	50.9	Sunny	1
	0.1	19.1	47.2	Sunny	0

### Data analysis

Of the four responses TP, FP, TN and FN, three parameters were calculated to evaluate the dogs detection performance in the final experiments^91^: correct responses among all responses, accuracy: (TP + TN) / (TP + TN + FP + FN), true positive responses among all target samples, sensitivity: TP / (TP + FN) and correct rejections of non-targets among all non-target samples: specificity: TN / (TN + FP). Accuracy express the percentage of correct responses (correctly identified targets and correctly rejected non-targets) among all possible responses (all scent samples in the experiment), while sensitivity express the percentage of correctly identified target scents among all target scents in the experiment and specificity express the percentage of correctly rejected non-targets among all non-target scents in the experiment.

### Ethical note

We carried out all work in accordance with the relevant guidelines and regulations of our university. The experimental protocol was approved by our animal ethical committee at the Department of Natural Sciences and Environmental Health, University of South-Eastern Norway*.* The study was also carried out in compliance with the ARRIVE guidelines^92^. Approvals from other ethics committees or ethics boards were not required. A written consent was not obtained from dog owners since both dogs are owned by the authors. Animals used in this study did not experience anesthesia, euthanasia or any kind of sacrifice.

## Supplementary Information

Supplementary Information 1.

Supplementary Information 2.

## Data Availability

Data used in this study will be available through the USN Research Data Archive.

## References

[1] IUCN. Guidlines for the prevention of biodiversity loss caused by alien invasive species. 1–24 (International Union for the Conservation of Nature, Switzerland, 2000).

[2] Wilcove DS, Rothstein D, Dubow J, Phillips A, Losos E (1998). Quantifying threats to imperiled species in the United States. Bio Sci..

[3] Pimentel D (2001). Economic and environmental threats of alien plant, animal, and microbe invasions. Agr. Ecosyst. Environ..

[4] Perrings C, Dehnen-Schmutz K, Touza J, Williamson M (2005). How to manage biological invasions under globalization. Trends Ecol. Evol..

[5] Baier SL, Bergstrand JH (2001). The growth of world trade: tariffs, transport costs, and income similarity. J. Int. Econ..

[6] Pimentel D, Zuniga R, Morrison D (2005). Update on the environmental and economic costs associated with alien-invasive species in the United States. Ecol. Econ..

[7] Xu H (2006). The distribution and economic losses of alien species invasion to China. Biol. Invasions.

[8] Meyerson LA, Mooney HA (2007). Invasive alien species in an era of globalization. Front. Ecol. Environ..

[9] Hulme PE (2008). Grasping at the routes of biological invasions: a framework for integrating pathways into policy. J. Appl. Ecol..

[10] Piel F, Gilbert M, De Cannière C, Grégoire JC (2008). Coniferous round wood imports from Russia and Baltic countries to Belgium. A pathway analysis for assessing risks of exotic pest insect introductions. Divers. Distrib..

[11] MacLeod A, Evans HF, Baker RHA (2002). An analysis of pest risk from an Asian longhorn beetle (*Anoplophora glabripennis*) to hardwood trees in the European community. Crop Prot..

[12] Liebhold AM, Brockerhoff EG, Garrett LJ, Parke JL, Britton KO (2012). Live plant imports: the major pathway for forest insect and pathogen invasions of the US. Front. Ecol. Environ..

[13] Pan, H. Y. Review of the Asian longhorned beetle: research, biology, distribution and management in China. (The General Station of Forest Pest Control The State Administration of Forestry, Shenyang, China, 2005).

[14] Lingafelter, S. W. & Hoebeke, E. R. *Revision of the genus Anoplophora (Coleoptera: Cerambycidae)*. (Entomological Society of Washington Washington, DC, 2002).

[15] Eschen R (2015). Trade patterns of the tree nursery industry in Europe and changes following findings of citrus longhorn beetle, *Anoplophora chinensis* Forster. NeoBiota.

[16] Sjöman H, Östberg J, Nilsson J (2014). Review of host trees for the wood-boring pests *Anoplophora glabripennis* and *Anoplophora chinensis*: an urban forest perspective. Arboric. Urban For..

[17] Cavey JF, Hoebeke ER, Passoa S, Lingafelter SW (1998). A new exotic threat to North American hardwood forests: an Asian longhorned beetle, *Anoplophora glabripennis* (Motschulsky) (Coleoptera: *Cerambycidae*).

[18] Haack RA, Hérard F, Sun J, Turgeon JJ (2010). Managing invasive populations of Asian longhorned beetle and Citrus longhorned beetle: a worldwide perspective. Annu. Rev. Entomol..

[19] Hérard F (2006). *Anoplophora* species in Europe: infestations and management processes. EPPO Bull..

[20] Hérard F (2009). Anoplophora glabripennis infestation (col: cerambycidae) in Italy. EPPO Bull..

[21] Nowak DJ, Pasek JE, Sequeira RA, Crane DE, Mastro VC (2001). Potential effect of *Anoplophora glabripennis* (*Coleoptera: Cerambycidae*) on urban trees in the United States. Econ. Entomol..

[22] European-Comission. Comission implementing decision of 1 of March 2012 as regards emergency measures to preven the introduction into and the spread within the Union of *Anoplophora chinensis* (Forster) *Official Journal of the European Union* **64**, 38–47 (2012).

[23] European-Comission. Comission implementing decision 2015/893 of 9 June 2015 as regards measures to prevent the introduction into and the spread within the Union of *Anoplophora glabirpennis* (Motschulsky). *Official Journal of the European Union* **146**, 16–28 (2015).

[24] USDA-APHIS. Asian Longhorned Beetle Eradication Program: Final Programmatic Environmental Impact Statement—September 2015. (USDA-APHIS, U.S, 2015).

[25] Keena M (2002). *Anoplophora glabripennis* (Coleoptera: Cerambycidae) fecundity and longevity under laboratory conditions: comparison of populations from New York and Illinois on *Acer saccharum*. Environ. Entomol..

[26] Meng PS, Hoover K, Keena MA (2015). Asian longhorned beetle (Coleoptera: *Cerambycidae*), an introduced pest of maple and other hardwood trees in North America and Europe. J. Integ. Pest Manag..

[27] Haack RA, Law KR, Mastro VC, Ossenburgen HS, Raimo BJ (1997). New York's battle with the Asian long-horned beetle. J. Forest..

[28] Hua, L., Li, S. & Zhang, X. in *Iconography of forest insects Hunan, China* (eds. J Peng & Y Liu) 467–524 (Hunan Scientifc and Technical Publishing House, 1992).

[29] Smith MJ, Turgeon JJ, De Groot P, Gasman B (2009). Asian longhorned beetle *Anoplophora glabripennis* (Motschulsky): lessons learned and opportunities to improve the process of eradication and management. Am. Entomol..

[30] Jin Y, Li J, Li J, Luo Y, Teale AS (2004). Olfactory response of *Anoplophora glabripennis* to volatile compounds from ash-leaf maple (*Acer negundo)* under drought stress. Scientia Silvae Sinicae.

[31] Zhang A, Oliver JE, Aldrich JR, Wang B, Mastro VC (2002). Stimulatory beetle volatiles for the Asian longhorned beetle, *Anoplophora glabripennis* (Motschulsky). Zeitschrift für Naturforschung C.

[32] Meng PS (2014). Effects of pheromone and plant volatile release rates and ratios on trapping *Anoplophora glabripennis* (Coleoptera: *Cerambycidae*) in China. Environ. Entomol..

[33] Mankin RW, Smith MT, Tropp JM, Atkinson EB, Jong DY (2008). Detection of *Anoplophora glabripennis* (Coleoptera: *Cerambycidae*) larvae in different host trees and tissues by automated analyses of sound-impulse frequency and temporal patterns. Econ. Entomol..

[34] Zorović M, Čokl A (2015). Laser vibrometry as a diagnostic tool for detecting wood-boring beetle larvae. Pest of Sci..

[35] Ric J (2006). Detecting Signs and Symptons of Asian Longhorned Beetle Injury: Training Guide.

[36] Hu J, Angeli S, Schuetz S, Luo Y, Hajek AE (2009). Ecology and management of exotic and endemic Asian longhorned beetle *Anoplophora glabripennis*. Agric. For. Entomol..

[37] Rosell F (2018). Secrets of the Snout: The Dog’s Incredible Nose.

[38] Suma P, La Pergola A, Longo S, Soroker V (2014). The use of sniffing dogs for the detection of *Rhynchophorus ferrugineus*. Phytoparasitica.

[39] Mosconi F (2017). Training of a dog for the monitoring of *Osmoderma eremita*. Nat. Conserv..

[40] Johansson A, Birgersson G, Schlyter F (2019). Using synthetic semiochemicals to train canines to detect bark beetle–infested trees. Ann. For. Sci..

[41] Hoyer-Tomiczek U, Sauseng G, Hoch G (2016). Scent detection dogs for the Asian longhorn beetle, *Anoplophora glabripennis*. EPPO Bull..

[42] Hoyer-Tomiczek, U. & Sauseng, G. in *Anoplophora chinensis & A. glabrinpennis: new tools for predicting, detecting and fighting. How to save our forests and our urban green spaces. **J. Entomolog. Acarolog. Res.* **45**, 10–12 (2013).

[43] Hoyer-Tomiczek U, Hoch G (2020). Progress in the use of detection dogs for emerald ash borer monitoring. Forestry.

[44] DeChant MT, Ford C, Hall NJ (2020). Effect of handler knowledge of the detection task on canine search behavior and performance. Front. Vet. Sci..

[45] Kardish MR (2015). Blind trust in unblinded observation in ecology, evolution, and behavior. Front. Ecol. Evol..

[46] Gadbois, S. & Reeve, C. in *Domestic dog dognition and behavior: the scientific study of Canis familiaris* (ed. Horowitz, A.) 3–29 (Springer, 2014).

[47] Minhinnick, S. in *Canine Olffaction Science and Law: Advances in Forensic Science, Medicine, Conservation and Environmental Remediation* (eds. Jezierski, T., Ensminger, J., & Papet, E.) (CRC Press Taylor and Francis Group, 2016).

[48] Schoon GAA (2002). Influence of experimental setup parameters of scent identification linups on their reliability. Problemy Kryminalistyki.

[49] Johnen D, Heuwieser W, Fischer-Tenhagen C (2017). An approach to identify bias in scent detection dog testing. Appl. Anim. Behav. Sci..

[50] Miklösi Á, Polgárdi R, Topál J, Csányi V (1998). Use of experimenter-given cues in dogs. Anim. Cogn..

[51] Lit L, Schweitzer JB, Oberbauer AM (2011). Handler beliefs affect scent detection dog outcomes. Anim. Cogn..

[52] Cooper JJ (2003). Clever hounds: social cognition in the domestic dog (*Canis familiaris*). Appl. Anim. Behav. Sci..

[53] Wasser SK (2004). Scat detection dogs in wildlife research and management: application to grizzly and black bears in the Yellowhead Ecosystem, Alberta, Canada. Can. J. Zool..

[54] Elliker KR (2014). Key considerations for the experimental training and evaluation of cancer odour detection dogs: lessons learnt from a double-blind, controlled trial of prostate cancer detection. BMC Urol..

[55] Moser A, Brown W, Bizo L, Andrew NR, Taylor M (2020). Biosecurity dogs detect live insects after training with odor-proxy training aids: scent extract and dead specimens. Chem. Senses.

[56] Lin H-M (2011). Fire ant-detecting canines: a complementary method in detecting red imported fire ants. J. Econ. Entomol..

[57] Cooper R, Wang C, Singh N (2014). Accuracy of trained canines for detecting bed bugs (Hemiptera: Cimicidae). J. Econ. Entomol..

[58] Pfiester M, Koehler P, Pereira R (2008). Ability of bed bug-detecting canines to locate live bed bugs and viable bed bug eggs. J. Econ. Entomol..

[59] Harrison RL (2006). From the field: a comparison of survey methods for detecting bobcats. Wildl. Soc. Bull..

[60] Reindl-Thompson SA, Shivik JA, Whitelaw A, Hurt A, Higgins KF (2006). Efficacy of scent dogs in detecting black-footed ferrets at a reintroduction site in South Dakota. Wildl. Soc. Bull..

[61] Cristescu RH (2015). Accuracy and efficiency of detection dogs: a powerful new tool for koala conservation and management. Sci. Rep..

[62] Mathews F (2013). Effectiveness of search dogs compared with human observers in locating bat carcasses at wind-turbine sites: a blinded randomized trial. Wildl. Soc. Bull..

[63] Long RA, Donovan TM, Mackay P, Zielinski WJ, Buzas JS (2007). Comparing scat detection dogs, cameras, and hair snares for surveying carnivores. J. Wildl. Manag..

[64] Williams M, Johnston JM (2002). Training and maintaining the performance of dogs (*Canis familiaris*) on an increasing number of odor discriminations in a controlled setting. Appl. Anim. Behav. Sci..

[65] Vynne C (2011). Effectiveness of scat-detection dogs in determining species presence in a tropical savanna landscape. Conserv. Biol..

[66] Long RA, Donovan TM, Mackay P, Zielinski WJ, Buzas JS (2007). Effectiveness of scat detection dogs for detecting forest carnivores. J. Wildl. Manag..

[67] Mazur JE (2016). Learning & Behavior.

[68] Orkin JD, Yang Y, Yang C, Douglas WY, Jiang X (2016). Cost-effective scat-detection dogs: unleashing a powerful new tool for international mammalian conservation biology. Sci. Rep..

[69] Beebe SC, Howell TJ, Bennett PC (2016). Using scent detection dogs in conservation settings: a review of scientific literature regarding their selection. Front. Vet. Sci..

[70] Hirai T (1996). Age-related changes in the olfactory system of dogs. Neuropathol. Apll. Neurobiol..

[71] Gadbois S, Reeve C (2016). The semiotic canine: scent processing dogs as research assistants in biomedical and environmental research. Dog Behav..

[72] Rosell F, Cross HB, Johnsen BC, Sundell J, Zedrosser A (2019). Scent-sniffing dogs can discriminate between native Eurasian and invasive North American beavers. Sci. Rep..

[73] Rosell F, Havier M, Kniha D (2020). Dogs can discriminate individual beavers from their anal gland secretion. Wildlife Biol..

[74] Arnesen HC, Johnsen BC, Costanzi J-M, Rosell F (2020). Canines (*Canis lupus familiaris*) as biodetectors for conservation work: can they discriminate the rock ptarmigan (*Lagopus muta*) from the willow grouse (*L. lagopus*) in a yes/no task?. PLoS ONE.

[75] Deldalle S, Gaunet F (2014). Effects of 2 training methods on stress-related behaviors of the dog (*Canis familiaris*) and on the dog–owner relationship. J. Vet. Beh.: Clin. Appl. Res..

[76] Chiandetti C, Avella S, Fongaro E, Cerri F (2016). Can clicker training facilitate conditioning in dogs?. Appl. Anim. Behav. Sci..

[77] Fjellanger R, Andersen EK, McLean I (2002). A training program for filter-search mine detection dogs. Int. J. Comp. Psychol..

[78] Jezierski, T. A. in *Canine olfaction sceince and law: advances in forensic science, medicine, conservation, and environmental remediation* (eds T.A. Jezierski, J.J. Ensminger, & L.E. Papet) Ch. 19, (CRC Press, 2016).

[79] Pfungst O, Rahn CL (1911). Clever Hans (the horse of Mr. Von Osten.): a Contribution to Experimental Animal and Human Psychology.

[80] Jezierski T (2014). Efficacy of drug detection by fully-trained police dogs varies by breed, training level, type of drug and search environment. Forensic Sci. Int..

[81] Traniello JF, Bakker TC (2015). Minimizing observer bias in behavioral research: blinded methods reporting requirements for behavioral ccology and sociobiology. Behav. Ecol. Sociobiol..

[82] Haack RA (2006). *Anoplophora glabripennis* within-tree distribution, seasonal development, and host suitability in China and Chicago. The Great Lakes Entomologist.

[83] Smith DA (2003). Detection and accuracy rates of dogs trained to find scats of San Joaquin kit foxes (*Vulpes macrotis mutica*). Anim. Conserv. Forum.

[84] Gazit I, Terkel J (2003). Domination of olfaction over vision in explosives detection by dogs. Appl. Anim. Behav. Sci..

[85] Glen, A. S. & Veltman, C. J. Search strategies for conservation detection dogs. *Wildlife Biology.* **1**, 1–9 (2018).

[86] Dahlgren, D. K. *et al.* in *Wildlife Techniques Manual* Vol. 1 (ed. N. Silvy) 140–153 (Wildlife Society Inc, 2012).

[87] Savidge JA, Stanford JW, Reed RN, Haddock GR, Adams AAY (2011). Canine detection of free-ranging brown treesnakes on Guam. N. Z. J. Ecol..

[88] Reed SE, Bidlack AL, Hurt A, Getz WM (2011). Detection distance and environmental factors in conservation detection dog surveys. J. Wildl. Manag..

[89] Glen AS, Russell JC, Veltman CJ, Fewster RM (2018). I smell a rat! Estimating effective sweep width for searches using wildlife-detector dogs. Wildl. Res..

[90] Jensen, I. S., Skaalin, R. & Eriksen, T. G. *Yr.no*, <https://www.yr.no/place/Norway/Vestfold_og_Telemark/Midt-Telemark/B%C3%B8~43270/> (2020).

[91] Jezierski T, Walczak M, Ligor T, Rudnicka J, Buszewski B (2015). Study of the art: canine olfaction used for cancer detection on the basis of breath odour. Perspectives and limitations. J. Breath Res..

[92] Percie du Sert N (2020). Reporting animal research: explanation and elaboration for the ARRIVE guidelines 2.0. PLOS Biol..

