# Supplementary material for: Pest detection dogs for wood boring longhorn beetles
Source: Sci Rep. 2021 Aug 19;11:16887. doi: 10.1038/s41598-021-96450-0 (PMC8376989; doi:10.1038/s41598-021-96450-0)
Supplement: Supplementary file 1 — Supplementary Information 1. [file 41598_2021_96450_MOESM1_ESM.pdf]

Appenix 1

Scent sample collection. Sample material = what kind of material the scent sample consisted from larvae. Host = what kind of host tree the sample material was provided from. Species = w the scent sample originated from. Weight = weight in grams (only frass or uninfested wood/ Provider = which company that provided the samples, or where the scent samples wer

Nr	Sample Material	Host	Species	Weight (g)
1	Frass (Larvae)	Populus tremula	Saperda populnea	0,640
2	Frass (Larvae)	Populus tremula	Saperda populnea	0,650
3	Frass (Larvae)	Populus tremula	Saperda populnea	0,690
4	Frass (Larvae)	Populus tremula	Saperda populnea	0,650
5	Frass (Larvae)	Populus tremula	Saperda populnea	0,710
6	Frass (Larvae)	Populus tremula	Saperda populnea	0,820
7	Frass (Larvae)	Populus tremula	Saperda populnea	0,690
8	Frass (Larvae)	Populus tremula	Saperda populnea	0,700
9	Frass (Larvae)	Populus tremula	Saperda populnea	0,590
10	Frass (Larvae)	Populus tremula	Saperda populnea	0,890
11	Frass (Larvae)	Populus tremula	Saperda populnea	0,950
12	Frass (Larvae)	Populus tremula	Saperda populnea	0,890
13	Frass (Larvae)	Populus tremula	Saperda populnea	0,640
14	Frass (Larvae)	Populus tremula	Saperda populnea	0,630
15	Frass (Larvae)	Populus tremula	Saperda populnea	0,720
16	Frass (Larvae)	Populus tremula	Saperda populnea	0,590
17	Frass (Larvae)	Populus tremula	Saperda populnea	0,810
18	Frass (Larvae)	Populus tremula	Saperda populnea	0,670
19	Frass (Larvae)	Populus tremula	Saperda populnea	0,760
20	Frass (Larvae)	Populus tremula	Saperda populnea	0,710
21	Frass (Larvae)	Populus tremula	Saperda populnea	0,810
22	Frass (Larvae)	Populus tremula	Saperda populnea	0,620
23	Frass (Larvae)	Populus tremula	Saperda populnea	0,630
24	Frass (Larvae)	Populus tremula	Saperda populnea	0,600
25	Frass (Larvae)	Populus tremula	Saperda populnea	0,620
26	Frass (Larvae)	Populus tremula	Saperda populnea	0,810
27	Frass (Larvae)	Populus tremula	Saperda populnea	0,590
28	Frass (Larvae)	Populus tremula	Saperda populnea	0,550
29	Frass (Larvae)	Populus tremula	Saperda populnea	0,670
30	Frass (Larvae)	Populus tremula	Saperda populnea	0,850
31	Frass (Larvae)	Populus tremula	Saperda populnea	0,790
32	Wood shaving	Populus tremula	Saperda populnea	2,432
33	Wood shaving	Populus tremula	Saperda populnea	2,111
34	Wood shaving	Populus tremula	Saperda populnea	2,328
35	Wood shaving	Populus tremula	Saperda populnea	2,486
36	Wood shaving	Populus tremula	Saperda populnea	2,571
37	Wood shaving	Populus tremula	Saperda populnea	2,553
38	Wood shaving	Populus tremula	Saperda populnea	2,194
39	Wood shaving	Populus tremula	Saperda populnea	2,313
40	Wood shaving	Populus tremula	Saperda populnea	2,774
41	Wood shaving	Populus tremula	Saperda populnea	2,473
42	Wood shaving	Populus tremula	Saperda populnea	2,382

43	Wood shaving	Populus tremula	Saperda populnea	2,385
44	Wood shaving	Populus tremula	Saperda populnea	2,595
45	Frass (Larvae)	Populus tremula	Saperda populnea	2,958
46	Frass (Larvae)	Populus tremula	Saperda populnea	2,384
47	Frass (Larvae)	Populus tremula	Saperda populnea	2,345
48	Wood shaving		*Acer platanoides*	2,183
49	Wood shaving		*Acer platanoides*	1,958
50	Wood shaving		*Acer platanoides*	1,495
51	Wood shaving		*Acer platanoides*	2,485
52	Wood shaving		*Acer platanoides*	2,394
53	Wood shaving		*Acer platanoides*	2,193
54	Wood shaving		*Acer platanoides*	2,460
55	Uninfested wood		*Acer platanoides*	2,450
56	Uninfested wood		*Acer platanoides*	2,540
57	Uninfested wood		*Acer platanoides*	2,304
58	Uninfested wood		*Acer platanoides*	2,740
59	Uninfested wood		*Acer platanoides*	2,340
60	Uninfested wood		*Acer platanoides*	2,320
61	Uninfested wood		*Acer platanoides*	2,432
62	Uninfested wood		*Acer platanoides*	2,394
63	Uninfested wood		*Acer platanoides*	2,847
64	Uninfested wood		*Acer platanoides*	2,503
65	Uninfested wood		*Acer platanoides*	2,009
66	Uninfested wood		*Acer platanoides*	2,496
67	Uninfested wood		*Acer platanoides*	2,424
68	Uninfested wood		*Acer platanoides*	2,496
69	Uninfested wood		*Acer platanoides*	1,499
70	Uninfested wood		*Acer platanoides*	1,394
71	Uninfested wood		*Acer platanoides*	1,984
72	Uninfested wood		*Acer platanoides*	1,394
73	Uninfested wood		*Acer platanoides*	2,303
74	Uninfested wood		*Acer platanoides*	2,405
75	Wood shaving		*Acer platanoides*	2,144
76	Wood shaving		*Acer platanoides*	2,401
77	Wood shaving		*Acer platanoides*	2,948
78	Wood shaving		*Acer platanoides*	2,884
79	Wood shaving		*Acer platanoides*	2,447
80	Wood shaving		*Acer platanoides*	2,400
81	Wood shaving		*Acer platanoides*	2,494
82	Wood shaving		*Acer platanoides*	2,392
83	Wood shaving		*Acer platanoides*	2,194
84	Uninfested wood		*Acer platanoides*	2,395
85	Wood shaving		*Salix caprea*	2,199
86	Wood shaving		*Salix caprea*	2,271
87	Wood shaving		*Salix caprea*	2,300
88	Wood shaving		*Salix caprea*	2,130

89	Wood shaving		*Salix caprea*	2,480
90	Wood shaving		*Salix caprea*	2,529
91	Wood shaving		*Salix caprea*	2,414
92	Wood shaving		*Salix caprea*	1,949
93	Wood shaving		*Salix caprea*	1,295
94	Wood shaving		*Salix caprea*	1,980
95	Uninfested wood		*Salix caprea*	1,298
96	Uninfested wood		*Salix caprea*	1,847
97	Uninfested wood		*Salix caprea*	2,984
98	Uninfested wood		*Salix caprea*	2,485
99	Uninfested wood		*Salix caprea*	2,382
100	Wood shaving		*Salix caprea*	1,295
101	Frass (Larvae)	Pinus sylvestris	*Rhagium inquisitor*	1,498
102	Frass (Larvae)	Pinus sylvestris	*Rhagium inquisitor*	2,484
103	Frass (Larvae)	Pinus sylvestris	*Rhagium inquisitor*	2,847
104	Frass (Larvae)	Pinus sylvestris	*Rhagium inquisitor*	2,486
105	Uninfested wood		*Salix caprea*	2,216
106	Uninfested wood		*Salix caprea*	2,155
107	Uninfested wood		*Salix caprea*	2,319
108	Uninfested wood		*Salix caprea*	2,138
109	Uninfested wood		*Salix caprea*	2,983
110	Frass (Larvae)	Pinus sylvestris	*Rhagium inquisitor*	2,413
111	Frass (Larvae)	Pinus sylvestris	*Rhagium inquisitor*	2,677
112	Frass (Larvae)	Pinus sylvestris	*Rhagium inquisitor*	2,343
113	Frass (Larvae)	Pinus sylvestris	*Rhagium inquisitor*	1,938
114	Frass (Larvae)	Pinus sylvestris	*Rhagium inquisitor*	1,894
115	Frass (Larvae)	Pinus sylvestris	*Rhagium inquisitor*	1,797
116	Frass (Larvae)	Pinus sylvestris	*Rhagium inquisitor*	2,584
117	Frass (Larvae)	Pinus sylvestris	*Rhagium inquisitor*	2,986
118	Frass (Larvae)	Pinus sylvestris	*Rhagium inquisitor*	2,578
119	Frass (Larvae)	Pinus sylvestris	*Rhagium inquisitor*	2,949
120	Frass (Larvae)	Pinus sylvestris	*Rhagium inquisitor*	1,995
121	Frass (Larvae)	*Pinus sylvestris*	*Rhagium inquisitor*	2,980
122	Frass (Larvae)	*Pinus sylvestris*	*Rhagium inquisitor*	2,174
123	Frass (Larvae)	*Pinus sylvestris*	*Rhagium inquisitor*	2,495
124	Frass (Larvae)	*Pinus sylvestris*	*Rhagium inquisitor*	2,194
125	Frass (Larvae)	*Pinus sylvestris*	*Rhagium inquisitor*	2,440
126	Frass (Larvae)	*Pinus sylvestris*	*Rhagium inquisitor*	2,949
127	Frass (Larvae)	*Pinus sylvestris*	*Rhagium inquisitor*	1,938
128	Frass (Larvae)	*Pinus sylvestris*	*Rhagium inquisitor*	1,989
129	Frass (Larvae)	*Pinus sylvestris*	*Rhagium inquisitor*	2,192
130	Frass (Larvae)	*Pinus sylvestris*	*Rhagium inquisitor*	2,114
131	Frass (Larvae)	*Pinus sylvestris*	*Rhagium inquisitor*	2,334
132	Frass (Larvae)	*Pinus sylvestris*	*Rhagium inquisitor*	2,844
133	Frass (Larvae)	*Pinus sylvestris*	*Rhagium inquisitor*	2,641
134	Frass (Larvae)	*Pinus sylvestris*	*Rhagium inquisitor*	1,948

135	Frass (Larvae)	*Pinus sylvestris*	*Rhagium inquisitor*	1,789
136	Larvae	*Pinus sylvestris*	*Rhagium inquisitor*	
137	Larvae	*Pinus sylvestris*	*Rhagium inquisitor*	
138	Larvae	*Pinus sylvestris*	*Rhagium inquisitor*	
139	Larvae	*Pinus sylvestris*	*Rhagium inquisitor*	
140	Larvae	*Pinus sylvestris*	*Rhagium inquisitor*	
141	Larvae	*Pinus sylvestris*	*Rhagium inquisitor*	
142	Larvae	*Pinus sylvestris*	*Rhagium inquisitor*	
143	Larvae	*Pinus sylvestris*	*Rhagium inquisitor*	
144	Larvae	*Pinus sylvestris*	*Rhagium inquisitor*	
145	Larvae	*Pinus sylvestris*	*Rhagium inquisitor*	
145	Larvae	*Pinus sylvestris*	*Rhagium inquisitor*	
147	Larvae	*Pinus sylvestris*	*Rhagium inquisitor*	
148	Larvae	*Pinus sylvestris*	*Rhagium inquisitor*	
149	Larvae	*Pinus sylvestris*	*Rhagium inquisitor*	
150	Larvae	*Pinus sylvestris*	*Rhagium inquisitor*	
151	Larvae	*Pinus sylvestris*	*Rhagium inquisitor*	
152	Larvae	*Pinus sylvestris*	*Rhagium inquisitor*	
153	Larvae	*Pinus sylvestris*	*Rhagium inquisitor*	
154	Larvae	*Pinus sylvestris*	*Rhagium inquisitor*	
155	Larvae	*Pinus sylvestris*	*Rhagium inquisitor*	
156	Larvae	*Pinus sylvestris*	*Rhagium inquisitor*	
157	Larvae	*Pinus sylvestris*	*Rhagium inquisitor*	
158	Larvae	*Pinus sylvestris*	*Rhagium inquisitor*	
159	Larvae	*Pinus sylvestris*	*Rhagium inquisitor*	
160	Larvae	*Pinus sylvestris*	*Rhagium inquisitor*	
161	Larvae	*Pinus sylvestris*	*Rhagium inquisitor*	
162	Larvae	*Pinus sylvestris*	*Rhagium inquisitor*	
163	Larvae	*Pinus sylvestris*	*Rhagium inquisitor*	
164	Larvae	*Pinus sylvestris*	*Rhagium inquisitor*	
165	Larvae	*Pinus sylvestris*	*Rhagium inquisitor*	
166	Larvae	*Pinus sylvestris*	*Rhagium inquisitor*	
167	Beetle	*Pinus sylvestris*	*Rhagium inquisitor*	
168	Beetle	*Pinus sylvestris*	*Rhagium inquisitor*	
169	Beetle	*Pinus sylvestris*	*Rhagium inquisitor*	
170	Beetle	*Pinus sylvestris*	*Rhagium inquisitor*	
171	Beetle	*Pinus sylvestris*	*Rhagium inquisitor*	
172	Beetle	*Pinus sylvestris*	*Rhagium inquisitor*	
173	Beetle	*Pinus sylvestris*	*Rhagium inquisitor*	
174	Beetle	*Pinus sylvestris*	*Rhagium inquisitor*	
175	Beetle	*Pinus sylvestris*	*Rhagium inquisitor*	
176	Beetle	*Pinus sylvestris*	*Rhagium inquisitor*	
177	Beetle	*Pinus sylvestris*	*Rhagium inquisitor*	
178	Beetle	*Pinus sylvestris*	*Rhagium inquisitor*	
179	Beetle	*Pinus sylvestris*	*Rhagium inquisitor*	
180	Beetle	*Pinus sylvestris*	*Rhagium inquisitor*	

181	Beetle	*Pinus sylvestris*	*Rhagium inquisitor*	
182	Pupae	*Pinus sylvestris*	*Rhagium inquisitor*	
183	Pupae	*Pinus sylvestris*	*Rhagium inquisitor*	
184	Pupae	*Pinus sylvestris*	*Rhagium inquisitor*	
185	Pupae	*Pinus sylvestris*	*Rhagium inquisitor*	
186	Pupae	*Pinus sylvestris*	*Rhagium inquisitor*	
187	Pupae	*Pinus sylvestris*	*Rhagium inquisitor*	
188	Beetle	*Pinus sylvestris*	*Monochamus stor*	
189	Beetle	*Pinus sylvestris*	*Monochamus stor*	
190	Beetle	*Pinus sylvestris*	*Monochamus stor*	
191	Beetle	*Pinus sylvestris*	*Monochamus stor*	
192	Beetle	*Pinus sylvestris*	*Monochamus stor*	
193	Beetle	*Pinus sylvestris*	*Monochamus stor*	
194	Beetle	*Pinus sylvestris*	*Monochamus stor*	
195	Beetle	*Pinus sylvestris*	*Monochamus stor*	
196	Beetle	*Pinus sylvestris*	*Monochamus stor*	
197	Beetle	*Pinus sylvestris*	*Monochamus stor*	
198	Beetle	*Pinus sylvestris*	*Monochamus stor*	
199	Beetle	*Pinus sylvestris*	*Monochamus stor*	
200	Larvae	*Pinus sylvestris*	*Monochamus stor*	
201	Larvae	*Pinus sylvestris*	*Monochamus stor*	
202	Larvae	*Pinus sylvestris*	*Monochamus stor*	
203	Larvae	*Pinus sylvestris*	*Monochamus stor*	
204	Larvae	*Pinus sylvestris*	*Monochamus stor*	
205	Larvae	*Pinus sylvestris*	*Monochamus stor*	
206	Larvae	*Pinus sylvestris*	*Monochamus stor*	
207	Larvae	*Pinus sylvestris*	*Monochamus stor*	
208	Larvae	*Pinus sylvestris*	*Monochamus stor*	
209	Larvae	*Pinus sylvestris*	*Monochamus stor*	
210	Larvae	*Pinus sylvestris*	*Monochamus stor*	
213	Frass (Larvae)	Salix alba	CLB	2,340
213	Frass (Larvae)	Salix alba	CLB	1,849
213	Frass (Larvae)	Salix alba	CLB	1,840
214	Frass (Larvae)	Salix alba	CLB	1,710
215	Frass (Larvae)	Salix alba	CLB	1,980
216	Frass (Larvae)	Salix alba	CLB	1,780
217	Frass (Larvae)	Salix alba	CLB	1,698
218	Frass (Larvae)	Salix alba	CLB	2,340
219	Frass (Larvae)	Salix alba	CLB	2,139
220	Frass (Larvae)	Salix alba	CLB	2,179
221	Frass (Larvae)	Salix alba	CLB	2,100
222	Frass (Larvae)	Salix alba	CLB	2,020
223	Frass (Larvae)	Salix alba	CLB	2,000
224	Frass (Larvae)	Salix alba	CLB	2,120
225	Frass (Larvae)	Salix alba	CLB	2,090
226	Frass (Larvae)	Salix alba	CLB	2,750

227	Frass (Beetle)	Acer negundo	CLB	2,640
228	Frass (Beetle)	Acer negundo	CLB	2,970
229	Frass (Beetle)	Acer negundo	CLB	2,670
230	Frass (Beetle)	Acer negundo	CLB	2,610
231	Frass (Beetle)	Acer negundo	CLB	2,760
232	Frass (Beetle)	Acer negundo	CLB	2,760
233	Frass (Beetle)	Acer negundo	CLB	2,870
234	Frass (Beetle)	Acer negundo	CLB	2,760
235	Frass (Beetle)	Acer negundo	CLB	2,700
236	Frass (Beetle)	Acer negundo	CLB	2,120
237	Frass (Beetle)	Acer negundo	CLB	2,670
238	Frass (Beetle)	Acer negundo	CLB	2,610
239	Frass (Beetle)	Acer negundo	CLB	2,760
240	Frass (Beetle)	Acer negundo	CLB	2,760
241	Frass (Beetle)	Acer negundo	CLB	2,870
242	Frass (Beetle)	Acer negundo	CLB	2,760
243	Frass (Beetle)	Acer negundo	CLB	2,700
244	Larvae	Acer pseudo	CLB	
245	Larvae	Acer pseudo	CLB	
246	Larvae	Acer pseudo	CLB	
247	Larvae	Acer pseudo	CLB	
248	Larvae	Acer pseudo	CLB	
249	Larvae	Acer pseudo	CLB	
250	Larvae	Salix alba	CLB	
251	Larvae	Salix alba	CLB	
252	Larvae	Salix alba	CLB	
253	Larvae	Salix alba	CLB	
254	Larvae	Salix alba	CLB	
255	Larvae	Salix alba	CLB	
256	Larvae	Salix alba	CLB	
257	Larvae	Salix alba	CLB	
258	Larvae	Salix alba	CLB	
259	Larvae	Salix alba	CLB	
260	Larvae	Salix alba	CLB	
261	Larvae	Salix alba	CLB	
262	Larvae	Salix alba	CLB	
263	Larvae	Salix alba	CLB	
264	Larvae	Salix alba	CLB	
265	Larvae	Salix alba	CLB	
266	Larvae	Salix alba	CLB	
267	Larvae	Salix alba	CLB	
268	Larvae	Salix alba	CLB	
269	Larvae	Salix alba	CLB	
270	Larvae	Salix alba	CLB	
271	Larvae	Salix alba	CLB	
272	Larvae	Salix alba	CLB	

273	Larvae	Salix alba	CLB
274	Larvae	Salix alba	CLB
275	Egg	Salix alba	CLB
276	Egg	Salix alba	CLB
277	Egg	Salix alba	CLB
278	Egg	Salix alba	CLB
279	Egg	Salix alba	CLB
280	Egg	Salix alba	CLB
281	Beetle	Salix alba & Acer negundo	CLB
282	Beetle	Salix alba & Acer negundo	CLB
283	Beetle	Salix alba & Acer negundo	CLB
284	Beetle	Salix alba & Acer negundo	CLB
285	Beetle	Salix alba & Acer negundo	CLB
286	Beetle	Salix alba & Acer negundo	CLB
287	Beetle	Salix alba & Acer negundo	CLB
288	Beetle	Salix alba & Acer negundo	CLB
289	Beetle	Salix alba & Acer negundo	CLB
290	Beetle	Salix alba & Acer negundo	CLB
291	Beetle	Salix alba & Acer negundo	CLB
292	Beetle	Salix alba & Acer negundo	CLB
293	Beetle	Salix alba & Acer negundo	CLB
294	Beetle	Salix alba & Acer negundo	CLB
295	Beetle	Salix alba & Acer negundo	CLB
296	Beetle	Salix alba & Acer negundo	CLB
297	Beetle	Salix alba & Acer negundo	CLB
298	Beetle	Salix alba & Acer negundo	CLB
299	Beetle	Salix alba & Acer negundo	CLB
300	Beetle	Salix alba & Acer negundo	CLB
301	Beetle	Salix alba & Acer negundo	CLB
302	Beetle	Salix alba & Acer negundo	CLB
303	Beetle	Salix alba & Acer negundo	CLB
304	Beetle	Salix alba & Acer negundo	CLB
305	Beetle	Salix alba & Acer negundo	CLB
306	Beetle	Salix alba & Acer negundo	CLB
307	Beetle	Salix alba & Acer negundo	CLB
308	Beetle	Salix alba & Acer negundo	CLB
309	Beetle	Salix alba & Acer negundo	ALB
310	Beetle	Salix alba & Acer negundo	ALB
311	Beetle	Salix alba & Acer negundo	ALB
312	Beetle	Salix alba & Acer negundo	ALB
313	Beetle	Salix alba & Acer negundo	ALB
314	Beetle	Salix alba & Acer negundo	ALB
315	Beetle	Salix alba & Acer negundo	ALB
316	Beetle	Salix alba & Acer negundo	ALB
317	Beetle	Salix alba & Acer negundo	ALB
318	Beetle	Salix alba & Acer negundo	ALB

319	Beetle	Salix alba & Acer negundo	ALB	
320	Frass (Beetle)	*Acer pensylvaticum*	ALB	2,190
321	Frass (Beetle)	*Acer pensylvaticum*	ALB	2,220
322	Frass (Beetle)	*Acer pensylvaticum*	ALB	2,080
323	Frass (Beetle)	*Acer pensylvaticum*	ALB	2,330
324	Frass (Beetle)	*Acer pensylvaticum*	ALB	2,110
325	Frass (Beetle)	*Acer pensylvaticum*	ALB	2,090
326	Frass (Beetle)	*Acer pensylvaticum*	ALB	2,080
327	Frass (Beetle)	*Acer pensylvaticum*	ALB	2,020
328	Frass (Beetle)	*Acer pensylvaticum*	ALB	2,430
329	Frass (Beetle)	*Acer pensylvaticum*	ALB	2,290
330	Frass (Beetle)	*Acer pensylvaticum*	ALB	2,220
331	Frass (Beetle)	*Acer pensylvaticum*	ALB	2,070
332	Frass (Beetle)	*Acer pensylvaticum*	ALB	2,090
333	Frass (Beetle)	*Acer pensylvaticum*	ALB	2,110
334	Frass (Beetle)	*Acer pensylvaticum*	ALB	2,110
335	Frass (Beetle)	*Acer pensylvaticum*	ALB	2,010
336	Frass (Beetle)	*Acer pensylvaticum*	ALB	2,410
337	Frass (Beetle)	*Acer pensylvaticum*	ALB	2,030
338	Frass (Beetle)	*Acer pensylvaticum*	ALB	2,170
339	Frass (Beetle)	*Acer pensylvaticum*	ALB	2,190
340	Frass (Beetle)	*Acer pensylvaticum*	ALB	2,080
341	Frass (Beetle)	*Acer pensylvaticum*	ALB	2,160
342	Frass (Beetle)	*Acer pensylvaticum*	ALB	2,630
343	Beetle	*Acer pensylvaticum*	ALB	
344	Beetle	*Acer pensylvaticum*	ALB	
345	Beetle	*Acer pensylvaticum*	ALB	
346	Beetle	*Acer pensylvaticum*	ALB	
347	Beetle	*Acer pensylvaticum*	ALB	
348	Beetle	*Acer pensylvaticum*	ALB	
349	Beetle	*Acer pensylvaticum*	ALB	
350	Beetle	*Acer pensylvaticum*	ALB	
351	Beetle	*Acer pensylvaticum*	ALB	
352	Beetle	*Acer pensylvaticum*	ALB	
353	Beetle	*Acer pensylvaticum*	ALB	
354	Beetle	*Acer pensylvaticum*	ALB	
355	Beetle	*Acer pensylvaticum*	ALB	
356	Beetle	*Acer pensylvaticum*	ALB	
357	Beetle	*Acer pensylvaticum*	ALB	
358	Beetle	*Acer pensylvaticum*	ALB	
359	Beetle	*Acer pensylvaticum*	ALB	
360	Beetle	*Acer pensylvaticum*	ALB	
361	Beetle	*Acer pensylvaticum*	ALB	
362	Beetle	*Acer pensylvaticum*	ALB	
363	Beetle	*Acer pensylvaticum*	ALB	
364	Beetle	*Acer pensylvaticum*	ALB	

365	Beetle	*Acer pensylvaticum*	ALB	
366	Beetle	*Acer pensylvaticum*	ALB	
367	Beetle	*Acer pensylvaticum*	ALB	
368	Beetle	*Acer pensylvaticum*	ALB	
369	Frass (Larvae)	*Acer platanoides*	ALB	2,070
370	Frass (Larvae)	*Acer platanoides*	ALB	2,090
371	Frass (Larvae)	*Acer platanoides*	ALB	2,030
372	Frass (Larvae)	*Acer platanoides*	ALB	2,160
373	Frass (Larvae)	*Acer platanoides*	ALB	2,160
374	Frass (Larvae)	*Acer platanoides*	ALB	2,190
375	Frass (Larvae)	*Acer platanoides*	ALB	2,090
376	Frass (Larvae)	*Acer platanoides*	ALB	2,050
377	Frass (Larvae)	*Acer platanoides*	ALB	2,190
378	Frass (Larvae)	*Acer platanoides*	ALB	2,190
379	Frass (Larvae)	*Acer platanoides*	ALB	2,080
380	Frass (Larvae)	*Acer platanoides*	ALB	2,250
381	Frass (Larvae)	*Acer platanoides*	ALB	2,490
382	Frass (Larvae)	*Acer platanoides*	ALB	2,020
383	Frass (Larvae)	*Acer platanoides*	ALB	2,070
384	Frass (Larvae)	*Acer platanoides*	ALB	2,160
385	Frass (Larvae)	*Acer platanoides*	ALB	2,111
386	Frass (Larvae)	*Acer platanoides*	ALB	2,380
387	Frass (Larvae)	*Acer platanoides*	ALB	2,030
388	Frass (Larvae)	*Acer platanoides*	ALB	2,020
389	Frass (Larvae)	*Acer platanoides*	ALB	2,170
390	Larvae	*Acer platanoides*	ALB	
391	Larvae	*Acer platanoides*	ALB	
392	Larvae	*Acer platanoides*	ALB	
393	Larvae	*Acer platanoides*	ALB	
394	Larvae	*Acer platanoides*	ALB	
395	Larvae	*Acer platanoides*	ALB	
396	Larvae	*Acer platanoides*	ALB	
397	Larvae	*Acer platanoides*	ALB	
398	Larvae	*Acer platanoides*	ALB	
399	Larvae	*Acer platanoides*	ALB	
400	Larvae	*Acer platanoides*	ALB	
401	Larvae	*Acer platanoides*	ALB	
402	Larvae	*Acer platanoides*	ALB	
403	Larvae	*Acer platanoides*	ALB	
404	Larvae	*Acer platanoides*	ALB	
405	Larvae	*Acer platanoides*	ALB	
406	Larvae	*Acer platanoides*	ALB	
407	Larvae	*Acer platanoides*	ALB	
408	Beetle	*Acer pensylvaticum*	ALB	
409	Beetle	*Acer pensylvaticum*	ALB	
410	Beetle	*Acer pensylvaticum*	ALB	

411	Beetle	*Acer pensylvaticum*	ALB
412	Beetle	*Acer pensylvaticum*	ALB
413	Beetle	*Acer pensylvaticum*	ALB
414	Beetle	*Acer pensylvaticum*	ALB
415	Beetle	*Acer pensylvaticum*	ALB
416	Beetle	*Acer pensylvaticum*	ALB
417	Beetle	*Acer pensylvaticum*	ALB
418	Beetle	*Acer pensylvaticum*	ALB
419	Beetle	*Acer pensylvaticum*	ALB
420	Beetle	*Acer pensylvaticum*	ALB

of e.g. frass made
hat kind of species
wood shavings).
e collected

Provider
Bø
Bø
Bø
Bø
Bø
Bø
Bø
Bø
Bø
Bø
Hvittingfoss
Hvittingfoss
Hvittingfoss
Hvittingfoss
Hvittingfoss
Hvittingfoss
Hvittingfoss
Hvittingfoss
Hvittingfoss
Hvittingfoss
Hvittingfoss
Hvittingfoss
Hvittingfoss
Hvittingfoss
Hvittingfoss
Hvittingfoss
Hvittingfoss
Hvittingfoss
Hvittingfoss
Hvittingfoss
Hvittingfoss
BØ
BØ
BØ
BØ
BØ
BØ
BØ
BØ
BØ
BØ
BØ

BØ
BØ
BØ
BØ
BØ
BØ
BØ
BØ
BØ
BØ
BØ
BØ
BØ
BØ
BØ
BØ
BØ
BØ
BØ
BØ
BØ
BØ
BØ
BØ
BØ
BØ
BØ
BØ
BØ
BØ
BØ
BØ
BØ
BØ
BØ
BØ
BØ
BØ
BØ
BØ
BØ
BØ
BØ
BØ
BØ
BØ

BØ
BØ
BØ
BØ
BØ
BØ
BØ
BØ
BØ
BØ
BØ
BØ
BØ
BØ
BØ
BØ
BØ
BØ
BØ
BØ
BØ
BØ
BØ
BØ
BØ
BØ
BØ
BØ
BØ
BØ
BØ
BØ
BØ
BØ
BØ
BØ
BØ
BØ
BØ
BØ
BØ
BØ
BØ
BØ
BØ
BØ

BØ
BØ
BØ
BØ
BØ
BØ
BØ
BØ
BØ
BØ
BØ
BØ
BØ
BØ
BØ
BØ
BØ
BØ
BØ
BØ
BØ
BØ
BØ
BØ
BØ
BØ
BØ
BØ
BØ
BØ
BØ
BØ
BØ
BØ
BØ
BØ
BØ
BØ
BØ
BØ
BØ
BØ
BØ
BØ
BØ
BØ

BØ
BØ
BØ
BØ
BØ
BØ
BØ
BØ
BØ
BØ
BØ
BØ
BØ
BØ
BØ
BØ
BØ
BØ
BØ
BØ
BØ
BØ
BØ
BØ
BØ
BØ
BØ
BØ
BØ
BØ
EBCL
EBCL
EBCL
EBCL
EBCL
EBCL
EBCL
EBCL
EBCL
EBCL
EBCL
EBCL
EBCL
EBCL
EBCL
EBCL

EBCL
EBCL
EBCL
EBCL
EBCL
EBCL
EBCL
EBCL
EBCL
EBCL
EBCL
EBCL
EBCL
EBCL
EBCL
EBCL
EBCL
EBCL
EBCL
EBCL
EBCL
EBCL
EBCL
EBCL
EBCL
EBCL
EBCL
EBCL
EBCL
EBCL
EBCL
EBCL
EBCL
EBCL
EBCL
EBCL
EBCL
EBCL
EBCL
EBCL
EBCL
EBCL
EBCL
EBCL
EBCL
EBCL

EBCL
EBCL
EBCL
EBCL
EBCL
EBCL
EBCL
EBCL
EBCL
EBCL
EBCL
EBCL
EBCL
EBCL
EBCL
EBCL
EBCL
EBCL
EBCL
EBCL
EBCL
EBCL
EBCL
EBCL
EBCL
EBCL
EBCL
EBCL
EBCL
EBCL
EBCL
EBCL
EBCL
EBCL
EBCL
EBCL
EBCL
EBCL
EBCL
EBCL
EBCL
EBCL
EBCL
EBCL
EBCL
EBCL

EBCL
IPS
IPS
IPS
IPS
IPS
IPS
IPS
IPS
IPS
IPS
IPS
IPS
IPS
IPS
IPS
IPS
IPS
IPS
IPS
IPS
IPS
IPS
IPS
IPS
IPS
IPS
IPS
IPS
IPS
IPS
IPS
IPS
IPS
IPS
IPS
IPS
IPS
IPS
IPS
IPS
IPS
IPS
IPS
IPS
IPS
IPS

IPS
IPS
IPS
IPS
APHIS US
APHIS US
APHIS US
APHIS US
APHIS US
APHIS US
APHIS US
APHIS US
APHIS US
APHIS US
APHIS US
APHIS US
APHIS US
APHIS US
APHIS US
APHIS US
APHIS US
APHIS US
APHIS US
APHIS US
APHIS US
APHIS US
APHIS US
APHIS US
APHIS US
APHIS US
APHIS US
APHIS US
APHIS US
APHIS US
APHIS US
APHIS US
APHIS US
APHIS US
APHIS US
APHIS US
APHIS US
APHIS US
APHIS US
APHIS US
APHIS US
APHIS US

APHIS US
APHIS US
APHIS US
APHIS US
APHIS US
APHIS US
APHIS US
APHIS US
APHIS US
APHIS US
