# Supplementary material for: Pest detection dogs for wood boring longhorn beetles
Source: Sci Rep. 2021 Aug 19;11:16887. doi: 10.1038/s41598-021-96450-0 (PMC8376989; doi:10.1038/s41598-021-96450-0)
Supplement: Supplementary file 2 — Supplementary Information 2. [file 41598_2021_96450_MOESM2_ESM.pdf]

Appendix 2

Scent sample collection. Sample material = what kind of material the scent sample consisted of e.g. frass made from larvae. Host = what kind of host tree the sample material was provided from. Species = what kind of species the scent sample originated from. Weight = weight in grams (only frass or uninfested wood/wood shavings). Provider = which company that provided the samples, or where the scent samples were collected

Nr	Experiment	Sample Material	Species	Weight (g)	Provider
30	1	Frass (Larvae)	Saperda populnea	0,850	Hvittingfoss
31	1	Frass (Larvae)	Saperda populnea	0,790	Hvittingfoss
45	1	Frass (Larvae)	Saperda populnea	2,958	BØ
46	1	Frass (Larvae)	Saperda populnea	2,384	BØ
47	1	Frass (Larvae)	Saperda populnea	2,345	BØ
52	1	Wood shaving	*Acer platanoides*	2,144	BØ
53	1	Wood shaving	*Acer platanoides*	2,401	BØ
54	1	Wood shaving	*Acer platanoides*	2,948	BØ
74	1	Uninfested wood	*Acer platanoides*	2,395	BØ
83	1	Wood shaving	*Salix caprea*	2,847	BØ
98	1	Uninfested wood	*Salix caprea*	2,949	BØ
99	1	Uninfested wood	*Salix caprea*	1,995	BØ
180	1	Beetle	*Rhagium inquisitor*		BØ
181	1	Beetle	*Rhagium inquisitor*		BØ
187	1	Pupae	*Rhagium inquisitor*		BØ
197	1	Beetle	*Monochamus stor*		BØ
198	1	Beetle	*Monochamus stor*		BØ
202	1	Larvae	*Monochamus stor*		BØ
203	1	Larvae	*Monochamus stor*		BØ
241	1	Frass (Beetle)	CLB	2,870	EBCL
242	1	Frass (Beetle)	CLB	2,760	EBCL
243	1	Frass (Beetle)	CLB	2,700	EBCL
272	1	Larvae	CLB		EBCL
273	1	Larvae	CLB		EBCL
274	1	Larvae	CLB		EBCL
307	1	Beetle	CLB		EBCL
308	1	Beetle	CLB		EBCL
318	1	Beetle	ALB		EBCL
319	1	Beetle	ALB		EBCL
364	1	Beetle	ALB		IPS
365	1	Beetle	ALB		IPS
366	1	Beetle	ALB		IPS
367	1	Beetle	ALB		IPS
368	1	Beetle	ALB		IPS
388	1	Frass (Larvae)	ALB	2,020	APHIS US
389	1	Frass (Larvae)	ALB	2,170	APHIS US
415	1	Beetle	ALB		APHIS US
24	2	Frass (Larvae)	Saperda populnea	0,600	Hvittingfoss
25	2	Frass (Larvae)	Saperda populnea	0,620	Hvittingfoss
26	2	Frass (Larvae)	Saperda populnea	0,810	Hvittingfoss

27	2	Frass (Larvae)	Saperda populnea	0,590	Hvittingfoss
28	2	Frass (Larvae)	Saperda populnea	0,550	Hvittingfoss
29	2	Frass (Larvae)	Saperda populnea	0,670	Hvittingfoss
48	2	Wood shaving	*Acer platanoides*	1,984	BØ
49	2	Wood shaving	*Acer platanoides*	1,394	BØ
50	2	Wood shaving	*Acer platanoides*	2,303	BØ
51	2	Wood shaving	*Acer platanoides*	2,405	BØ
71	2	Uninfested wood	*Acer platanoides*	2,494	BØ
72	2	Uninfested wood	*Acer platanoides*	2,392	BØ
73	2	Uninfested wood	*Acer platanoides*	2,194	BØ
79	2	Wood shaving	*Salix caprea*	2,382	BØ
80	2	Wood shaving	*Salix caprea*	1,295	BØ
81	2	Wood shaving	*Salix caprea*	1,498	BØ
82	2	Wood shaving	*Salix caprea*	2,484	BØ
96	2	Uninfested wood	*Salix caprea*	2,986	BØ
97	2	Uninfested wood	*Salix caprea*	2,578	BØ
121	2	Frass (Larvae)	*Rhagium inquisitor*	2,980	BØ
122	2	Frass (Larvae)	*Rhagium inquisitor*	2,174	BØ
123	2	Frass (Larvae)	*Rhagium inquisitor*	2,495	BØ
124	2	Frass (Larvae)	*Rhagium inquisitor*	2,194	BØ
125	2	Frass (Larvae)	*Rhagium inquisitor*	2,440	BØ
126	2	Frass (Larvae)	*Rhagium inquisitor*	2,949	BØ
160	2	Larvae	*Rhagium inquisitor*		BØ
161	2	Larvae	*Rhagium inquisitor*		BØ
162	2	Larvae	*Rhagium inquisitor*		BØ
163	2	Larvae	*Rhagium inquisitor*		BØ
164	2	Larvae	*Rhagium inquisitor*		BØ
165	2	Larvae	*Rhagium inquisitor*		BØ
166	2	Larvae	*Rhagium inquisitor*		BØ
170	2	Beetle	*Rhagium inquisitor*		BØ
176	2	Beetle	*Rhagium inquisitor*		BØ
177	2	Beetle	*Rhagium inquisitor*		BØ
178	2	Beetle	*Rhagium inquisitor*		BØ
179	2	Beetle	*Rhagium inquisitor*		BØ
186	2	Pupae	*Rhagium inquisitor*		BØ
194	2	Beetle	*Monochamus stor*		BØ
195	2	Beetle	*Monochamus stor*		BØ
196	2	Beetle	*Monochamus stor*		BØ
200	2	Larvae	*Monochamus stor*		BØ
201	2	Larvae	*Monochamus stor*		BØ
222	2	Frass (Larvae)	CLB	2,020	EBCL
223	2	Frass (Larvae)	CLB	2,000	EBCL
224	2	Frass (Larvae)	CLB	2,120	EBCL
225	2	Frass (Larvae)	CLB	2,090	EBCL
226	2	Frass (Larvae)	CLB	2,750	EBCL
238	2	Frass (Beetle)	CLB	2,610	EBCL

239	2	Frass (Beetle)	CLB	2,760	EBCL
240	2	Frass (Beetle)	CLB	2,760	EBCL
265	2	Larvae	CLB		EBCL
266	2	Larvae	CLB		EBCL
267	2	Larvae	CLB		EBCL
268	2	Larvae	CLB		EBCL
269	2	Larvae	CLB		EBCL
270	2	Larvae	CLB		EBCL
271	2	Larvae	CLB		EBCL
278	2	Egg	CLB		EBCL
279	2	Egg	CLB		EBCL
303	2	Beetle	CLB		EBCL
304	2	Beetle	CLB		EBCL
305	2	Beetle	CLB		EBCL
306	2	Beetle	CLB		EBCL
314	2	Beetle	ALB		EBCL
315	2	Beetle	ALB		EBCL
316	2	Beetle	ALB		EBCL
317	2	Beetle	ALB		EBCL
339	2	Frass (Beetle)	ALB	2,190	IPS
340	2	Frass (Beetle)	ALB	2,080	IPS
341	2	Frass (Beetle)	ALB	2,160	IPS
342	2	Frass (Beetle)	ALB	2,630	IPS
360	2	Beetle	ALB		IPS
361	2	Beetle	ALB		IPS
362	2	Beetle	ALB		IPS
363	2	Beetle	ALB		IPS
384	2	Frass (Larvae)	ALB	2,160	APHIS US
385	2	Frass (Larvae)	ALB	2,111	APHIS US
386	2	Frass (Larvae)	ALB	2,380	APHIS US
387	2	Frass (Larvae)	ALB	2,030	APHIS US
404	2	Larvae	ALB		APHIS US
405	2	Larvae	ALB		APHIS US
406	2	Larvae	ALB		APHIS US
407	2	Larvae	ALB		APHIS US
416	2	Beetle	ALB		APHIS US
417	2	Beetle	ALB		APHIS US
6	3	Frass (Larvae)	Saperda populnea	0,820	Bø
7	3	Frass (Larvae)	Saperda populnea	0,690	Bø
8	3	Frass (Larvae)	Saperda populnea	0,700	Bø
9	3	Frass (Larvae)	Saperda populnea	0,590	Bø
10	3	Frass (Larvae)	Saperda populnea	0,890	Bø
22	3	Frass (Larvae)	Saperda populnea	0,620	Hvittingfoss
23	3	Frass (Larvae)	Saperda populnea	0,630	Hvittingfoss
55	3	Uninfested wood	*Acer platanoides*	2,884	BØ
56	3	Uninfested wood	*Acer platanoides*	2,447	BØ

57	3	Uninfested wood	*Acer platanoides*	2,400	BØ
75	3	Wood shaving	*Salix caprea*	1,298	BØ
76	3	Wood shaving	*Salix caprea*	1,847	BØ
77	3	Wood shaving	*Salix caprea*	2,984	BØ
78	3	Wood shaving	*Salix caprea*	2,485	BØ
84	3	Uninfested wood	*Salix caprea*	2,486	BØ
95	3	Uninfested wood	*Salix caprea*	2,584	BØ
100	3	Frass (Larvae)	*Rhagium inquisitor*	1,789	BØ
101	3	Frass (Larvae)	*Rhagium inquisitor*	1,359	BØ
102	3	Frass (Larvae)	*Rhagium inquisitor*	2,496	BØ
103	3	Frass (Larvae)	*Rhagium inquisitor*	2,469	BØ
104	3	Frass (Larvae)	*Rhagium inquisitor*	2,996	BØ
116	3	Frass (Larvae)	*Rhagium inquisitor*	1,949	BØ
117	3	Frass (Larvae)	*Rhagium inquisitor*	1,989	BØ
118	3	Frass (Larvae)	*Rhagium inquisitor*	1,900	BØ
119	3	Frass (Larvae)	*Rhagium inquisitor*	1,789	BØ
120	3	Frass (Larvae)	*Rhagium inquisitor*	1,927	BØ
136	3	Larvae	*Rhagium inquisitor*		BØ
137	3	Larvae	*Rhagium inquisitor*		BØ
138	3	Larvae	*Rhagium inquisitor*		BØ
139	3	Larvae	*Rhagium inquisitor*		BØ
140	3	Larvae	*Rhagium inquisitor*		BØ
141	3	Larvae	*Rhagium inquisitor*		BØ
142	3	Larvae	*Rhagium inquisitor*		BØ
143	3	Larvae	*Rhagium inquisitor*		BØ
144	3	Larvae	*Rhagium inquisitor*		BØ
145	3	Larvae	*Rhagium inquisitor*		BØ
159	3	Larvae	*Rhagium inquisitor*		BØ
167	3	Beetle	*Rhagium inquisitor*		BØ
168	3	Beetle	*Rhagium inquisitor*		BØ
169	3	Beetle	*Rhagium inquisitor*		BØ
185	3	Pupae	*Rhagium inquisitor*		BØ
199	3	Beetle	*Monochamus stor*		BØ
204	3	Larvae	*Monochamus stor*		BØ
219	3	Frass (Larvae)	CLB	2,139	EBCL
220	3	Frass (Larvae)	CLB	2.179	EBCL
221	3	Frass (Larvae)	CLB	2,100	EBCL
235	3	Frass (Beetle)	CLB	2,700	EBCL
236	3	Frass (Beetle)	CLB	2,120	EBCL
237	3	Frass (Beetle)	CLB	2,670	EBCL
247	3	Larvae	CLB		EBCL
248	3	Larvae	CLB		EBCL
249	3	Larvae	CLB		EBCL
263	3	Larvae	CLB		EBCL
264	3	Larvae	CLB		EBCL
280	3	Egg	CLB		EBCL

300	3	Beetle	CLB		EBCL
301	3	Beetle	CLB		EBCL
302	3	Beetle	CLB		EBCL
309	3	Beetle	ALB		EBCL
310	3	Beetle	ALB		EBCL
311	3	Beetle	ALB		EBCL
312	3	Beetle	ALB		EBCL
313	3	Beetle	ALB		EBCL
332	3	Frass (Beetle)	ALB	2,090	IPS
333	3	Frass (Beetle)	ALB	2,110	IPS
334	3	Frass (Beetle)	ALB	2,110	IPS
335	3	Frass (Beetle)	ALB	2,010	IPS
336	3	Frass (Beetle)	ALB	2,410	IPS
337	3	Frass (Beetle)	ALB	2,030	IPS
338	3	Frass (Beetle)	ALB	2,170	IPS
356	3	Beetle	ALB		IPS
357	3	Beetle	ALB		IPS
358	3	Beetle	ALB		IPS
359	3	Beetle	ALB		IPS
380	3	Frass (Larvae)	ALB	2,250	APHIS US
381	3	Frass (Larvae)	ALB	2,490	APHIS US
382	3	Frass (Larvae)	ALB	2,020	APHIS US
383	3	Frass (Larvae)	ALB	2,070	APHIS US
399	3	Larvae	ALB		APHIS US
400	3	Larvae	ALB		APHIS US
401	3	Larvae	ALB		APHIS US
402	3	Larvae	ALB		APHIS US
403	3	Larvae	ALB		APHIS US
418	3	Beetle	ALB		APHIS US
419	3	Beetle	ALB		APHIS US
420	3	Beetle	ALB		APHIS US
